# Regularizing and Normalizing DAGs and Phylogenetic Networks

**DOI:** 10.1007/s11538-026-01783-4

**Published:** 2026-10-21

**Authors:** Marc Hellmuth, Anna Lindeberg, Vincent Moulton

**Affiliations:** 1 Faculty of Mathematics and Computer Science, Leipzig University, 04109 Leipzig, Germany; 2 Department of Mathematics, Faculty of Science, Stockholm University, SE-10691 Stockholm, Sweden; 3 School of Computing Sciences, University of East Anglia, Norwich, NR4 7TJ UK

**Keywords:** Phylogenetic network, Regular network, Normal network, Cluster, Least common ancestors

## Abstract

Phylogenetic networks and, more generally, directed acyclic graphs (DAGs) represent hierarchical structure beyond trees, for instance in the presence of reticulate evolutionary events such as hybridization or horizontal gene transfer. A central question is which parts of such graphs are essential with respect to leaf-observable information, and which parts can be removed without changing this information. Resolving this question can lead to principled simplification methods for phylogenetic networks, such as the recent normalization approach of Francis et al. In this paper, we study this question from three related perspectives: clusters displayed by a DAG *G*, least common ancestors (LCAs) of subsets of its leaf set, and visibility, a path-based property of vertices. We first introduce an LCA-based simplification procedure called *i-regularization*. For a DAG *G* and i≥1, the DAG $\textsc {Reg}_i(G)$ retains precisely those vertices that occur as unique LCAs of leaf subsets of size at most *i*, removes the remaining non-leaf vertices by a graph-editing operation ⊖, and then deletes shortcuts. We show that $\textsc {Reg}_i(G)$ admits a Hasse-diagram characterization in terms of the corresponding lca-clusters. We then compare LCA-based regularization with normalization. Using the same ⊖-operator, we describe the cover construction underlying normalization, identify visible vertices that are nevertheless removed, and characterize when regularization and normalization coincide. Together, these results provide a unified framework for cluster-based, LCA-based, and visibility-based simplifications of DAGs and phylogenetic networks.

## Introduction

Directed acyclic graphs (DAGs) and rooted phylogenetic networks are widely used to model hierarchical structure in situations where purely tree-like representations are too restrictive. A classical example arises in evolutionary biology, where reticulation events such as hybridization or horizontal gene transfer cannot be captured by trees alone (Huson and Scornavacca 2011). Beyond phylogenetics, DAG-based models also appear in areas such as database theory (Aho et al. 1981), workflow analysis and process modeling (van der Aalst 1998, 2004), or in the theory of Bayesian and causal networks (Pearl 2009; Koller and Friedman 2009).

A recurring theme in the study of such graphs is to determine which aspects of the underlying topology can be recovered from “coarse” information derived from the leaves, and how one can systematically simplify a given DAG or network without destroying the leaf-implied structure of interest. In phylogenetics, the leaves represent present-day species (or other taxa), while internal vertices encode ancestral ones. It is therefore natural to ask which internal vertices are structurally essential and which are redundant with respect to a specified type of leaf-based information.

**Fig. 1:**
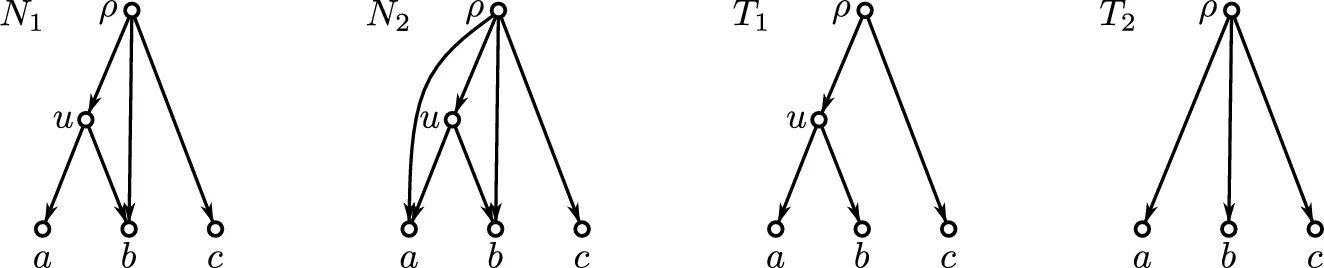
Two networks $N_1$, $N_2$ and two trees $T_1$, $T_2$, for which $T_1 =\textsc {Reg}(N_1) = \textsc {Norm}(N_1)$ and $T_1=\textsc {Reg}(N_2)\ne \textsc {Norm}(N_2)=T_2$, see Sect. 3

Three classical viewpoints on this question are central to this paper. The first is *cluster information*: every vertex *v* in a DAG *G* with leaf set *X* induces a cluster ${{\,\mathrm{\texttt{C}}\,}}_G(v) = \{x\in X \mid \, \text {there is a directed path from } v \text { to } x \text { in }G\}$, and the resulting set system $\mathfrak {C}(G)$ records how leaves are grouped by ancestral vertices. The second is *least common ancestors (LCAs)*: for a subset A⊆X, its least common ancestors (when they exist) identify the “lowest” vertices that jointly explain the ancestry of *A*. The third is *visibility*, a path-based notion that captures whether a vertex lies on all root-to-leaf paths leading to at least one leaf. For example, in Fig. 1, taking *G* to be the DAG $N_1$, the vertex *u* induces the cluster $\{a,b\}$, the LCA of the vertices *a* and *c* is ρ, and the vertex *u* is visible, since all paths from the root ρ to the leaf *a* pass through *u*.

This leads to a fundamental question: given a DAG or network together with its induced leaf-based information – such as clusters, LCAs, or visibility – which structural components are essential for representing this information, and which are superfluous? In other words, can certain internal vertices be removed or suitably transformed to yield a simpler directed graph that still faithfully captures the prescribed leaf-based structure? From this perspective, simplifying a DAG or network means defining a principled transformation that eliminates “irrelevant” substructures – those that do not contribute to the chosen leaf-based information – while preserving the structural relationships among the leaves. The main challenge is to formalize this notion of irrelevance and to design simplification procedures that are canonical, well-behaved, and compatible with the underlying leaf-based representation.

In Francis et al. (2021), Francis, Huson and Steel introduced a *normalization* procedure that transforms a network *N* into a normal network $\textsc {Norm}(N)$ by retaining only visible vertices, removing shortcuts, and suppressing vertices of in-degree and out-degree equal to one. More recently, Lindeberg and Hellmuth (2025, 2026) proposed an alternative simplification approach based on retaining only those vertices that serve as least common ancestors for specified subsets of leaves. A third, conceptually related, construction is to consider the Hasse diagram of the clustering system $\mathfrak {C}(G)$, which yields a canonical DAG that is minimal in the sense of representing all leaf-induced clusters. Note that there are other approaches for simplifying DAGs and networks. Some focus on producing a tree from a given DAG or network, see for example (Gusfield 2014; Huber et al. 2019; Dress et al. 2010; Heiss et al. 2025; Willson 2011; Baroni et al. 2006), and further simplifications that do not necessarily produce a tree are presented in Moret et al. (2004); Willson (2012, 2022).

Although the normalization, the LCA-driven and the Hasse diagram approaches produce a simplified version of the underlying DAG or network – essentially by removing vertices deemed “irrelevant” and rewiring certain arcs – each of them relies on different criteria. Normalization is based on visibility and thus on path-based properties, whereas the LCA-driven simplification retains vertices according to their role in explaining leaf subsets. The relationship between a DAG *G* and the Hasse diagram $\mathcal {H}(\mathfrak {C}(G))$ of its cluster system has also been partially explored in Lindeberg and Hellmuth (2025); Hellmuth and Lindeberg (2026); Baroni et al. (2005); in essence, $\mathcal {H}(\mathfrak {C}(G))$ retains single vertices *v* for which ${{\,\mathrm{\texttt{C}}\,}}_G(v)$ is contained in $\mathfrak {C}(G)$ and its arcs reflect how such clusters are related with respect to set inclusion. Our main contribution is to highlight the similarities and differences between these three approaches to simplifying DAGs and networks, and to place them within a unified conceptual framework.

In the following, we begin with the LCA-driven perspective and specialize the simplification framework originally introduced in Lindeberg and Hellmuth (2025) to a systematic “regularization” approach based on the notion of *lca-relevance*. More specifically, for each integer i≥1, we consider the set of vertices that arise as the unique LCA, denoted $\operatorname {lca}_G(A)$, for some subset A⊆X with |A|≤i, and we delete all other non-leaf vertices using the graph-editing operator ⊖ (which generalizes suppression and certain arc contractions). After subsequently removing shortcuts (denoted with a superscripted “−”), this yields the *i-regularization* $\textsc {Reg}_i(G) =(G\ominus W)^-$ of *G*. Intuitively, $\textsc {Reg}_i(G)$ is a canonical simplification of *G* that preserves the LCA structure of all leaf subsets of size at most *i*. Moreover, $\textsc {Reg}_i(G)$ is *regular*, which means that it is completely determined by its set of clusters.

Although regularity of $\textsc {Reg}_i(G)$ is an immediate consequence of results in Lindeberg and Hellmuth (2025), that paper does not describe the set of clusters in $\mathfrak {C}(\textsc {Reg}_i(G))$. To complete the picture, we give a cluster-level characterization of *i*-regularization in Theorem 3.5. Specifically, we show that $\textsc {Reg}_i(G)$ is isomorphic to the Hasse diagram of certain *lca-clusters*: clusters *C* for which $\operatorname {lca}_G(C)$ is well-defined and forced by some subset *A* with |A|≤i. Thus, Theorem 3.5 provides a direct link between LCA-based simplification and the classical cluster/Hasse-diagram viewpoint. This is complemented by Theorem 3.8, which characterizes when the Hasse diagram $\mathcal {H}(\mathfrak {C}(G))$ coincides with the fully regularized graph $\textsc {Reg}(G):=\textsc {Reg}_{|X|}(G)$.

Next, we consider normal networks and their behavior under regularization. In Proposition 3.11, we extend the connection between normal and regular networks established in Theorem 3.9 of Willson (2010) by clarifying the relationships between normality, strong normality, lca-relevance, and regularity. We then show in Theorem 3.14 precisely how regularization acts on a normal network. Besides providing further structural insight into normal networks, these results provide key ingredients to characterize when normalization and regularization yield the same network, cf. Theorem 4.6.

We then turn to the principal goal of this paper: comparing regularization with the normalization $\textsc {Norm}(N)$ of *N*. These two approaches are distinct. For example, for the networks $N_1$ and $N_2$ in Fig. 1, the networks $\textsc {Reg}(N_1)$ and $\textsc {Norm}(N_1)$ coincide, whereas the networks $\textsc {Reg}(N_2)$ and $\textsc {Norm}(N_2)$ do not. Although both approaches aim to remove “irrelevant” vertices, they differ precisely in the criterion used to determine which vertices are retained: normalization is based on visibility, a path-based notion that captures whether a vertex lies on all root-to-leaf paths to some leaf, whereas *i*-regularization is driven by lca-relevance.

To better understand the interplay between these two notions of relevance, in Sect. 4 we express both constructions within the common framework of transformations of the form $(N\ominus W)^-$. The key difference lies in the choice of the vertex set *W*. For *i*-regularization, *W* consists, by definition, of the vertices that are not unique LCAs of leaf subsets of size at most *i*. For normalization, we first show that the cover construction $\textsc {Cov}(N)$ can be expressed as $\textsc {Cov}(N)=(N\ominus \overline{\mathfrak {vis}}(N))^-$, where $\overline{\mathfrak {vis}}(N)$ is the set of non-visible vertices of *N*. We then identify a subclass of visible vertices that are suppressed in the second step of normalization, the so-called *dispensably visible* vertices, and provide criteria for detecting them directly in *N*. This yields an ⊖-based characterization of normalization itself and, ultimately, allows us to characterize precisely when $\textsc {Reg}_i(N)$ and $\textsc {Norm}(N)$ coincide; see Theorem 4.6, which is the main comparison result of the paper.

The rest of this paper is organized as follows. In Sect. 2 we recall the required background on DAGs, shortcuts, normal networks, the ⊖-operator, clusters, and LCAs. Section 3 introduces *i*-regularization, discusses its basic properties, and characterizes $\textsc {Reg}_i(G)$ via lca-clusters and Hasse diagrams. It also discusses normal and strongly normal networks and relates their structure to lca-relevance. In particular, we describe how normal networks behave under *i*-regularization, that is, under the transformation $N\mapsto \textsc {Reg}_i(N)$. In Sect. 4 we then develop the framework for the comparison of network regularization and normalization, derive ⊖-based formulas for $\textsc {Cov}(N)$ and $\textsc {Norm}(N)$, and identify conditions under which regularization and normalization agree. Finally, in Sect. 5 we conclude with a discussion of some future directions.

## Preliminaries

In this section, we recall the main definitions and results concerning DAGs, LCAs, clusters, and the ⊖-operator that are needed throughout the paper. Most of this material is taken from, or follows directly from, Lindeberg and Hellmuth (2025) and related work; it is included here for reference and to fix notation. Those readers who are more familiar with these concepts can move on to the next section and refer back to the relevant statements if necessary.

**Basics.** In what follows, *X* will always be a finite non-empty set and $\mathscr {P}_{\le i}(X)$ the set of non-empty subsets of *X* of size at most *i*. Moreover,  denotes the *disjoint union* of two sets *A* and *B*. A collection $\mathfrak {T}$ of non-empty subsets of *X* is a *hierarchy* if $X\in \mathfrak {T}$, $\{x\}\in \mathfrak {T}$ for all x∈X, and for all $A,B\in \mathfrak {T}$, we have $A\cap B\in \{\emptyset ,A,B\}$.

A *directed graph *G=(V,E) is an ordered tuple with non-empty vertex set V(G):=V and arc set $E(G):=E\subseteq V\times V$. We sometimes use u→v to refer to the arc (u,v)∈E(G) and u⇝v to denote a directed *uv*-path in *G*. We put ${{\,\textrm{outdeg}\,}}_G(v):=\left| \left\{ u\in V :(v,u)\in E\right\} \right|$ and ${{\,\textrm{indeg}\,}}_G(v):=\left| \left\{ u\in V :(u,v)\in E\right\} \right|$ to denote the *out-degree* and *in-degree* of a vertex *v*, respectively. A vertex *v* with ${{\,\textrm{outdeg}\,}}_G(v)=0$ is a *leaf* of *G* and a vertex *v* with ${{\,\textrm{indeg}\,}}_G(v)=0$ is a *root* of *G*. A *hybrid* is a vertex *v* with ${{\,\textrm{indeg}\,}}_G(v)\ge 2$. Note that leaves might also be hybrids. A directed graph *G* is *phylogenetic* if it does not contain a vertex *v* such that ${{\,\textrm{outdeg}\,}}_G(v)=1$ and ${{\,\textrm{indeg}\,}}_G(v)\le 1$.

Directed graphs *G* without directed cycles are called *directed acyclic graphs (DAGs)*. If *G* is a DAG with leaf set *X*, then *G* is a *DAG on X*. A *network* is a DAG with a unique root. A *(rooted) tree* is a network that does not contain any hybrid vertices. A DAG *G* is *separated* if all hybrids *v* in *G* have ${{\,\textrm{outdeg}\,}}_G(v)=1$ (Hellmuth et al. 2023).

Let *G* be a DAG. We write $v\preceq _G w$ if and only if there is a directed *wv*-path in *G*. If $v\preceq _G w$ and v≠w, we write $v\prec _G w$. If u→v is an arc in *G*, then $v\prec _G u$ and we call *v* a *child* of *u* and *u* a *parent* of *v*. In a DAG *G* where $u\preceq _G v$, we call *u* a *descendant* of *v* and *v* an *ancestor* of *u*. If $u\preceq _G v$ or $v\preceq _G u$, then *u* and *v* are $\preceq _G$*-comparable* and, otherwise, $\preceq _G$*-incomparable*.

Two DAGs *G* and *H* are *isomorphic* if there is an isomorphism between *G* and *H*, i.e., a bijective map $\varphi :V(G)\rightarrow V(H)$ such that (u,v)∈E(G) if and only if $(\varphi (u),\varphi (v))\in E(H)$. If this is the case, we write G≃H.

An arc e=(u,w) in a DAG *G* is a *shortcut* if there is a directed *uw*-path that does not contain the arc *e* (Linz and Semple 2020; Döcker et al. 2019). A DAG without shortcuts is *shortcut-free*. If F⊆E is the set of all shortcuts of G=(V,E), then we put $G^-:=(V,E\setminus F)$.

### Lemma 2.1

((Lindeberg and Hellmuth, 2025, L. 2.5) and (Willson, 2016,L. 7.2)) Let *G* be a DAG on *X*. Then, $G^-$ is a shortcut-free DAG on *X* such that, for all u,v∈V(G), we have $u \preceq _{G} v$ if and only if $u \preceq _{G^-} v$. Moreover, if *G* and *H* are shortcut-free DAGs such that V:=V(G)=V(H) and, for all u,v∈V, it holds that $u\preceq _G v$ if and only if $u\preceq _H v$, then G=H.

**Normal Networks.** Normal networks form a prominent class of phylogenetic networks (Francis et al. 2021; Willson 2010); see Francis (2025) for a comprehensive overview. Although our primary focus lies on normal *networks*, several technical results are more naturally formulated in the broader setting of DAGs. For this reason, we develop the theory at the level of DAGs and specialize to networks where appropriate.

### Definition 2.2

A child *u* of *v* in a DAG *G* is a *tree-child* if ${{\,\textrm{indeg}\,}}_G(u) = 1$. A DAG *G* on *X* is *tree-child* if every vertex $v \in V(G) \setminus X$ has a tree-child (Cardona et al. 2009). A DAG is *normal* if it is tree-child and shortcut-free (Willson 2010).

A common restriction in the phylogenetic literature, in particular for normal networks, is that hybrid vertices are required to have a unique child. We explicitly call such networks *separated*. To avoid some technical issues caused by separated vertices, we introduce the following class of DAGs:

### Definition 2.3

A DAG is *strongly normal* if it is normal and does not contain any vertices that have only one child.

Let *N* be a network on *X* with unique root ρ. A vertex *v* of *N* is *visible (in N)* if there exists a leaf x∈X such that every ρx-path of *N* passes through *v*. Let $\mathfrak {vis}(N)$ denote the set of visible vertices of *N* and put $\overline{\mathfrak {vis}}(N):=V(N){\setminus } \mathfrak {vis}(N)$. Observe that, by definition, ρ and every x∈X are visible vertices of *N*. The following result provides an interesting characterization of normal networks.

### Theorem 2.4

(Cardona et al., 2009,Lem. 2) A network is normal if and only if it is shortcut-free and all its vertices are visible.

**The **⊖**-operator.** We continue by summarizing important concepts from Lindeberg and Hellmuth (2025) where, in particular, the following ⊖-operator is studied.

### Definition 2.5

(Shanavas et al. (2024)) Let G=(V,E) be a DAG and v∈V. Then $G⊖v=(V^{\prime },E^{\prime })$ is the directed graph with vertex set $$V'=V\setminus \{v\}$$ and arcs $\left(p,q\right)\in E^{\prime }$ precisely if v≠p, v≠q and (p,q)∈E, or if (p,v)∈E and (v,q)∈E. For a non-empty subset $$W = \{w_1,\dots ,w_\ell \} \subsetneq V$$, define $$G\ominus W :=(\dots ((G \ominus w_1) \ominus w_2) \dots )\ominus w_\ell $$. For notational reasons, put $$G\ominus \emptyset =G$$.

In simple terms, the directed graph G⊖v is obtained from G=(V,E) by deleting the vertex *v* together with all incident arcs, and then adding an arc p→q for every parent *p* of *v* and every child *q* of *v*. If *v* is a leaf or a root, we simply remove *v* and its incident arcs. If *v* has in-degree one and out-degree one in *G*, then G⊖v is well-known as the directed graph obtained from *G* by *suppressing* *v*. More generally, if *v* has a unique child *u*, then G⊖v can be viewed as contracting the arc v→u to the single vertex *u*. Similarly, if *v* has a unique *parent* *u*, then G⊖v coincides with contracting the arc u→v to *u*. Hence, the ⊖-operator unifies and generalizes several common graph-editing operations on DAGs or networks.

It is also worth noting that the definition of G⊖W is motivated by the fact that the ⊖-operator is commutative. This fact is mentioned –– without proof –– in Shanavas et al. (2024); Lindeberg and Hellmuth (2025). For completeness, we shall prove this in the appendix of the paper.

### Lemma 2.6

For all DAG *G* and distinct vertices u,v∈V(G) it holds that $(G \ominus v) \ominus u = (G \ominus u) \ominus v$.

### Proof

The proof is straightforward but tedious and, therefore, outsourced to Appendix A. □

Hence, the order of the vertices in *W* does not affect the result of $G \ominus W :=(\dots ((G \ominus w_1) \ominus w_2) \dots ) \ominus w_\ell$. It is easily seen that G⊖W remains a DAG and that the existence of directed paths in *G* is preserved in G⊖W. The following lemma summarizes these simple but important properties of the ⊖-operator.

### Lemma 2.7

(Lindeberg and Hellmuth, 2025,Obs 5.3) Let *G* be a DAG on *X* and $W \subseteq V (G) \setminus X$. Then, G⊖W is a DAG on *X* that satisfies $u\preceq _{G} v\iff u\preceq _{G\ominus W} v$ for all $u,v \in V (G \ominus W)=V(G){\setminus } W$.

Let *G* be a DAG on *X*. For every v∈V(G) we define the *cluster* of *v* as the set ${{\,\mathrm{\texttt{C}}\,}}_G(v):=\{x\in X\mid x\preceq _G v\}$. The set $\mathfrak {C}(G)=\{{{\,\mathrm{\texttt{C}}\,}}_G(v)\mid v\in V(G)\}$ comprises all clusters of the vertices in *G*.

Just as every DAG *G* on *X* naturally gives rise to a set system on *X*, we can also associate to every set system on *X* a corresponding DAG on *X*, as we now explain. The *Hasse diagram* $\mathcal {H}(\mathfrak {C})$ of a set system $\mathfrak {C}$ on *X* is the DAG with vertex set $\mathfrak {C}$ and arc set containing an arc A→B for $A,B\in \mathfrak {C}$ if and only if (i) B⊊A and (ii) there is no $C\in \mathfrak {C}$ with $B\subsetneq C\subsetneq A$. The Hasse diagram $\mathcal {H}(\mathfrak {C})$ is also known as the *cover digraph* of $\mathfrak {C}$ (Baroni et al. 2005). Intuitively, DAGs that are indistinguishable from the Hasse diagram of their set of clusters have much of their structure determined by these clusters. Such DAGs are called *regular*, and are formally defined as follows.

### Definition 2.8

(Baroni et al. (2005)) A DAG G=(V,E) is *regular* if the map $\varphi :V\rightarrow V(\mathcal {H}(\mathfrak {C}(G)))$ defined by $v\mapsto {{\,\mathrm{\texttt{C}}\,}}_G(v)$ is an isomorphism between *G* and $\mathcal {H}(\mathfrak {C}(G))$.

Note that regular DAGs have been shown to be the only DAGs that can arise from certain simple models of evolution, see Baroni et al. (2005); Baroni and Steel (2006) for details. As we shall see, regular DAGs are also closely related to properties involving so-called least common ancestors, which we define next. For a given DAG *G* on *X* and a non-empty subset A⊆X, a vertex v∈V(G) is a *common ancestor of A* if $A\subseteq {{\,\mathrm{\texttt{C}}\,}}_G(v)$, i.e., if *v* is an ancestor of every vertex in *A*. Moreover, *v* is a *least common ancestor* (LCA) of *A* if *v* is $\preceq _G$-minimal among all common ancestors of *A*. The set $\operatorname {LCA}_G(A)$ comprises all LCAs of *A* in *G*.

**Fig. 2:**
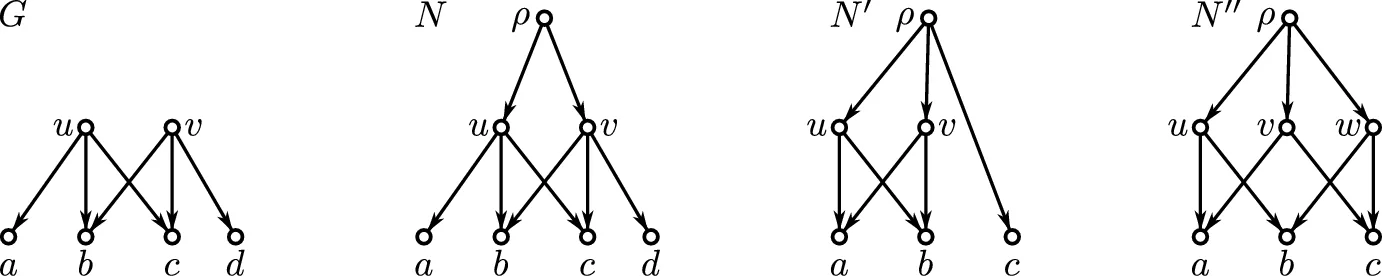
Four DAGs that illustrate certain concepts related to least common ancestors; see the text for further details

In general, not every set A⊆X has a (unique) least common ancestor in a DAG; for example, consider the DAG *G* in Fig. 2 where $|\operatorname {LCA}_G(\{b,c\})|>1$ and $\operatorname {LCA}_G(\{a,d\})=\emptyset$. In a network *N* on *X*, the unique root is a common ancestor for all A⊆X and, therefore, $\operatorname {LCA}_N(A)\ne \emptyset$. For simplicity, we will write $\operatorname {lca}_G(A)=v$ in case that $\operatorname {LCA}_G(A)=\{v\}$, in which case we say that $\operatorname {lca}_G(A)$ *is well-defined*; otherwise, we leave $\operatorname {lca}_G(A)$ *undefined*. Moreover, when $\operatorname {lca}_G(\{x,y\})$ is well-defined for some x,y∈X, we write $\operatorname {lca}_G(x,y)$ to refer to $\operatorname {lca}_G(\{x,y\})$. A vertex v∈V(G) is a *k-lca vertex* if $v=\operatorname {lca}_G(A)$ for some A⊆X with |A|=k. In addition, a DAG *G* satisfies the *cluster-lca (CL)* property if $\operatorname {lca}_G({{\,\mathrm{\texttt{C}}\,}}_G(v))$ is well-defined for all v∈V(G).

Not all DAGs satisfy the (CL) property. For example, in the network $N^{\prime }$ of Fig. 2, $$\operatorname {LCA}_{N'}({{\,\mathrm{\texttt{C}}\,}}_{N'}(v))=\operatorname {LCA}_{N'}(\{a,b\})=\{u,v\}$$ and thus, $$\operatorname {lca}_{N'}({{\,\mathrm{\texttt{C}}\,}}_{N'}(v))$$ is not well-defined. Nevertheless, as the following result shows, $$\operatorname {lca}_G({{\,\mathrm{\texttt{C}}\,}}_G(v))$$ is well-defined whenever $$v=\operatorname {lca}_G(A)$$ for some subset A⊆X. This, and the other properties stated in the following lemma, are simple consequences of the definitions and the fact that $${{\,\mathrm{\texttt{C}}\,}}_G(u)\subseteq {{\,\mathrm{\texttt{C}}\,}}_G(v)$$ for all u,v∈V(G) such that $$u\preceq _G v$$.

### Lemma 2.9

((Shanavas et al., 2024,Obs. 2) & (Lindeberg and Hellmuth, 2025,Cor. 3.6)) If *G* is a DAG on *X* and A⊆X is a non-empty subset such that $\operatorname {lca}_G(A)$ is well-defined, then ${{\,\mathrm{\texttt{C}}\,}}_G(\operatorname {lca}_G(A))$ is the unique inclusion-minimal cluster in $\mathfrak {C}(G)$ containing *A*, and $\operatorname {lca}_G({{\,\mathrm{\texttt{C}}\,}}_G(\operatorname {lca}_G(A)))=\operatorname {lca}_G(A)$. In particular, v∈V(G) is a *k*-lca vertex in *G* for some *k* if and only if $v = \operatorname {lca}_G ({{\,\mathrm{\texttt{C}}\,}}_G (v))$. Moreover, if u∈V(G) is a vertex such that $A\subseteq {{\,\mathrm{\texttt{C}}\,}}_G(u)$, then $\operatorname {lca}_G(A)\preceq _G u$.

Note that, if *w* is the unique child of a vertex *v* in a DAG *G*, then ${{\,\mathrm{\texttt{C}}\,}}_G(v)={{\,\mathrm{\texttt{C}}\,}}_G(w)$ holds. This together with Lemma 3.4 and 3.5 in Lindeberg and Hellmuth (2025) implies the following natural property of vertices with out-degree one.

### Lemma 2.10

If *v* is a vertex of a DAG *G* on *X* such that ${{\,\textrm{outdeg}\,}}_G(v)=1$, then $v\notin \operatorname {LCA}_G(A)$ for any A⊆X.

The following definition from Lindeberg and Hellmuth (2025) captures the notion of a DAG where every vertex is a *k*-lca vertex for some k≤i.

### Definition 2.11

A DAG G=(V,E) on *X* is *i*-lca-relevant if, for all v∈V, there is some $A\in \mathscr {P}_{\le i}(X)$ such that $v = \operatorname {lca}_G(A)$.

In other words, *G* is *i*-lca-relevant precisely if every vertex of *G* is a *k*-lca vertex for some k≤i. Hence, if *G* is *i*-lca-relevant for some integer *i*, then *G* is also *j*-lca-relevant for all j≥i. As shown by the DAG $N^{\prime \prime }$ in Fig. 2, the converse of the latter statement is not always satisfied: $N^{\prime \prime }$ is 3-lca-relevant, but not 2-lca-relevant since its root ρ is not the unique LCA for any subset consisting of one or two leaves only. Note also that the DAG *G* in Fig. 2 is 2-lca-relevant, as $$\operatorname {lca}_G(x)=x$$ for all $$x\in \{a,b,c,d\}$$, and $$u=\operatorname {lca}_G(a,b)$$, $$v=\operatorname {lca}_G(c,d)$$ are all well-defined. This holds even though $$\operatorname {lca}_G(b,c)$$ is undefined. We call |*X*|-lca-relevant DAGs on *X* simply *lca-relevant* since, in this case, each vertex *v* is the unique LCA of just *some* subset of its leaves, with no restriction on the size of that set. By way of example, the DAGs *G*, *N* and $N^{\prime \prime }$ of Fig. 2 are lca-relevant, while the network $N^{\prime }$ is not. Clearly, all *i*-lca-relevant DAGs are, in particular, lca-relevant.

We also note that there is a surprisingly close connection between regular and lca-relevant DAGs:

### Theorem 2.12

(Lindeberg and Hellmuth, 2025,Thm. 4.10) A DAG *G* is regular if and only if *G* is lca-relevant and shortcut-free.

We close this section by collecting together a couple of results that connect the ⊖-operator with clusters and LCAs and that we shall need later on. The first observation is a direct consequence of Lemma 2.1 and Lemma 2.7.

### Observation 2.13

Let G=(V,E) be a DAG on *X* and $W\subseteq V\setminus X$. Then, $H=(G\ominus W)^-$ is a shortcut-free DAG on *X* that satisfies $u\preceq _G v \iff u\preceq _H v$ for all $u,v\in V(H)=V{\setminus } W$. Consequently, ${{\,\mathrm{\texttt{C}}\,}}_H(v)={{\,\mathrm{\texttt{C}}\,}}_G(v)$ for all $v\in V{\setminus } W$ and, thus, $\mathfrak {C}(H)=\{{{\,\mathrm{\texttt{C}}\,}}_G(v)\mid v\in V\setminus W \}\subseteq \mathfrak {C}(G)$.

In case *W* solely contains vertices of out-degree one, we have further control over properties of G⊖W.

### Lemma 2.14

Let G=(V,E) be a DAG on *X* and let $W\subseteq \{v\in V\mid {{\,\textrm{outdeg}\,}}_G(v)=1\}$. Then G⊖W is a DAG on *X* such that

1. $\operatorname {LCA}_{G\ominus W}(A)=\operatorname {LCA}_G(A)$ for all non-empty A⊆X and
2. $$1\le {{\,\textrm{outdeg}\,}}_{G\ominus W}(v)\le {{\,\textrm{outdeg}\,}}_G(v)$$ for all $$v\in V(G\ominus W) {\setminus } X$$.

In particular, if $W'\subseteq \{w\in W\mid \text {the unique child } c \text { of } w \text { satisfies } {{\,\textrm{indeg}\,}}_G(c)=1\},$ then ${{\,\textrm{outdeg}\,}}_{G\ominus W'}(v)={{\,\textrm{outdeg}\,}}_G(v)$ for all $v\in V(G\ominus W')$.

### Proof

Let *G* and *W* be as stated. Since every vertex in *W* has out-degree one, *W* contains no leaf of *G*. Hence, Lemma 2.7 implies that G⊖W is again a DAG on *X*.

For Statement (1), note that if w∈W, then $w\notin \operatorname {LCA}_G(Y)$ for any Y⊆X, see Lemma 2.10. This together with (Lindeberg and Hellmuth, 2025, Thm 5.5) ensures that $\operatorname {LCA}_{G\ominus W}(A)=\operatorname {LCA}_G(A)$ for all non-empty A⊆X.

We next prove Statement (2). Consider first a single vertex w∈W, and let *c* be its unique child. By Definition 2.5, passing from the current DAG *G* to the graph G⊖w can only affect the out-degree of a parent *p* of *w*. By construction of G⊖w, *w* is removed from ${{\,\textrm{child}\,}}_G(p)$ and *c* is added as a child of *p* unless *c* is already a child of *p*. Hence the out-degree of *p* either remains unchanged or decreases by one; in either case *c* is a child of *p* in G⊖w. This observation can be applied successively to the vertices of *W*. Indeed, every vertex w∈W that has not yet been removed still has out-degree one: if its unique child is removed at an earlier step, that child is replaced by its unique child. Consequently, $1\le {{\,\textrm{outdeg}\,}}_{G\ominus W}(v)\le {{\,\textrm{outdeg}\,}}_G(v)$ for every $v\in V(G\ominus W) \setminus X$.

Finally, let $$ W'\subseteq \{w\in W\mid \text {the unique child }$$c$$\text { of }$$w$$\text { satisfies } {{\,\textrm{indeg}\,}}_G(c)=1\}$$. We prove the claim by induction on $|W^{\prime }|$. The assertion is trivial for $W^{\prime }=\emptyset$. Suppose now that $$W'\ne \emptyset $$ and that the claim holds for every proper subset of $W^{\prime }$. Since *G* is acyclic and every vertex of $W^{\prime }$ has out-degree one, there exists some $w\in W^{\prime }$ whose unique child *c* does not belong to $W^{\prime }$. Put $$ S:=W'{\setminus }\{w\}$$ and H:=G⊖S. By the induction hypothesis, $$ {{\,\textrm{outdeg}\,}}_H(v)={{\,\textrm{outdeg}\,}}_G(v) $$ for every v∈V(H). In particular, $$ {{\,\textrm{outdeg}\,}}_H(w)=1$$. Since w,c∉S, the vertex *c* and the arc w→c is still present in *H*. Hence *c* is the unique child of *w* in *H*. Moreover, $${{\,\textrm{indeg}\,}}_G(c)=1$$, with *w* being the unique parent of *c* in *G*. Any arc p→c in H=G⊖S corresponds to a directed *pc*-path in *G* whose internal vertices belong to *S*. Since the last arc of every such path must be w→c and w∉S, this is only possible if p=w. Consequently, $$ {{\,\textrm{indeg}\,}}_H(c)=1$$. Now let *p* be any parent of *w* in *H*. Since *w* is the unique parent of *c*, we have (p,c)∉E(H). Thus, by Definition 2.5, passing from *H* to H⊖w replaces the child *w* of *p* by the child *c* and therefore preserves the out-degree of *p*. The out-degrees of all other remaining vertices are unaffected. Hence $$ {{\,\textrm{outdeg}\,}}_{H\ominus w}(v)={{\,\textrm{outdeg}\,}}_H(v) $$ for every $$v\in V(H\ominus w)$$. Since $$ H\ominus w=(G\ominus S)\ominus w=G\ominus W'$$, we obtain $$ {{\,\textrm{outdeg}\,}}_{G\ominus W'}(v)={{\,\textrm{outdeg}\,}}_G(v) $$ for every $$v\in V(G\ominus W')$$. □

The condition on $W^{\prime }$ in Lemma 2.14 is sufficient to ensure that out-degrees are preserved, but cannot easily be omitted. Indeed, suppose that a vertex *v* has a child *c* that is also a child of a parent *p* of *v*. Then removing *v* via the ⊖-operator reduces the out-degree of *p* by one, so $$ {{\,\textrm{outdeg}\,}}_{G\ominus v}(p)={{\,\textrm{outdeg}\,}}_G(p)-1$$. Even in a shortcut-free DAG, two vertices of out-degree one may have the same parent and the same child. Removing both vertices then decreases the out-degree of their common parent by one. Thus, shortcut-freeness alone does not guarantee preservation of out-degrees under G⊖W.

In the final definition for this section, we provide a slight generalization of regular DAGs.

### Definition 2.15

(Hellmuth et al. (2023)) A DAG is *semi-regular* if it is shortcut-free and satisfies the following condition

(PCC): for all u,v∈V(G) it holds that *u* and *v* are $\preceq _G$-comparable if and only if ${{\,\mathrm{\texttt{C}}\,}}_G(u) \subseteq {{\,\mathrm{\texttt{C}}\,}}_G(v)$ or ${{\,\mathrm{\texttt{C}}\,}}_G(v) \subseteq {{\,\mathrm{\texttt{C}}\,}}_G(u)$.

Theorem 4.6 of Lindeberg and Hellmuth (2025) shows that semi-regular DAGs generalize regular DAGs, i.e., that every regular DAG is also semi-regular. As noted earlier, the DAGs *G*, *N*, and $N^{\prime \prime }$ in Fig. 2 are regular, and thus also semi-regular. In contrast, the network $N^{\prime }$ is not semi-regular: it contains the $$\preceq _{N'}$$-incomparable vertices *u* and *v* that satisfy $${{\,\mathrm{\texttt{C}}\,}}_{N'}(u)=\{a,b\}={{\,\mathrm{\texttt{C}}\,}}_{N'}(v)$$. Therefore, $N^{\prime }$ violates the (PCC) property.

## Regularizations

In this section, we consider a systematic way to transform a DAG into a regular DAG while preserving its least common ancestor (LCA) structure up to some prescribed level. The regularization procedure considered here is a specialization of the more general LCA-based simplification framework introduced in Lindeberg and Hellmuth (2025). We formulate this construction explicitly in terms of subsets of bounded cardinality, which leads to the notion of *i*-regularization. Intuitively, this amounts to removing vertices that do not play any role as LCAs of “small” subsets of leaves.

In Sect. 3.1, we recall the basic properties of this construction and derive new connections between *i*-regularization, lca-clusters, and Hasse diagrams. In particular, we characterize the set of clusters of $\textsc {Reg}_i(G)$ in terms of lca-clusters and show that $\textsc {Reg}_i(G)$ is isomorphic to the Hasse diagram of this cluster system (see Theorem 3.5). We further characterize when the fully regularized DAG $\textsc {Reg}(G)$ coincides with the Hasse diagram of the complete cluster system $\mathfrak {C}(G)$ (see Theorem 3.8).

In Sect. 3.2, we investigate how LCA-based regularization acts on normal networks and, in particular, clarify the relationship between normality, strong normality, and lca-relevance. We show that if *N* is a normal network, then $\textsc {Reg}(N)$ is again a normal network, and characterize when $\textsc {Reg}(N)=N$ (see Theorem 3.14). These structural results also provide important ingredients for Sect. 4, where we compare regularization with normalization within the common ⊖-framework.

### Regularizing DAGs

Recall that $\mathscr {P}_{\le i}(X)$ denotes the non-empty subsets of *X* of size at most *i*. For an integer i≥1 and a DAG *G*, define the set

$$ \begin{aligned} \mathfrak {lca}_{i}(G)&:=\left\{ v\in V(G)\mid v=\operatorname {lca}_G(A) \text { for some } A\in \mathscr {P}_{\le i}(X)\right\} \text { and }\\ &\quad \overline{\mathfrak {lca}_{i}}(G) :=V(G){\setminus } \mathfrak {lca}_{i}(G). \end{aligned} $$

In other words, $\mathfrak {lca}_{i}(G)$ contains each vertex of *G* that is a *k*-lca vertex for some k≤i while $\overline{\mathfrak {lca}_{i}}(G)$ is the complement of that set. Note that $X\subseteq \mathfrak {lca}_{i}(G)$ for all *i*, since every x∈X satisfies $x=\operatorname {lca}_G(\{x\})$ for any DAG *G* on *X*.

Another simple observation stems from Lemma 2.10, namely, if a vertex *v* has out-degree one, then it is not an LCA of *any* set of leaves. Consequently, vertices of out-degree one are in particular always elements of $\overline{\mathfrak {lca}_{i}}(G)$ for any integer *i* and any DAG *G*. The next lemma gives a slight extension of this result which we will use later.

#### Lemma 3.1

Suppose *G* is a DAG on *X* and let $W=\{w\in V(G)\mid {{\,\textrm{outdeg}\,}}_G(w)=1\}$. Then,

$$ \begin{aligned} X&=\mathfrak {lca}_{1}(G)\subseteq \mathfrak {lca}_{2}(G)\subseteq \ldots \subseteq \mathfrak {lca}_{|X|}(G)\subseteq V(G){\setminus } W,&\text {and}\\ W&\subseteq \overline{\mathfrak {lca}_{|X|}}(G)\subseteq \ldots \subseteq \overline{\mathfrak {lca}_{2}}(G)\subseteq \overline{\mathfrak {lca}_{1}}(G)=V(G){\setminus } X&\end{aligned} $$

#### Proof

Let *G* be a DAG on *X*, |X|=ℓ and $W=\{w\in V(G)\mid {{\,\textrm{outdeg}\,}}_G(w)=1\}$. Note that $\mathfrak {lca}_{1}(G)=X$ follows from the fact that $v=\operatorname {lca}_G(\{x\})$ for some x∈X if and only if v=x. Moreover, the inclusions $\mathfrak {lca}_{i}(G)\subseteq \mathfrak {lca}_{i+1}(G)$ for each $1\le i< \ell$ follow from the definition: if $v\in \mathfrak {lca}_{i}(G)$, then *v* is a *j*-lca vertex for some *j* with $1\le j\le i$ and thus clearly also for some *j* with $1\le j\le i+1$, i.e. $v\in \mathfrak {lca}_{i+1}(G)$. Moreover, Lemma 2.10 implies that $w\notin \operatorname {LCA}_G(A)$ for all w∈W and non-empty A⊆X. Consequently, $\mathfrak {lca}_{\ell }(G)\cap W=\emptyset$. Therefore,

$$X=\mathfrak {lca}_{1}(G)\subseteq \mathfrak {lca}_{2}(G)\subseteq \ldots \subseteq \mathfrak {lca}_{\ell }(G)\subseteq V(G){\setminus } W.$$

The second chain of inclusions is now an immediate consequence of the latter statement and the fact that $\overline{\mathfrak {lca}_{i}}(G)=V(G)\setminus \mathfrak {lca}_{i}(G)$ for all *i*. □

We now introduce the main definition of this section. It defines a transformation that maps a DAG *G* to a DAG $G^{\prime }$ by first removing, via the ⊖-operator, all vertices that are not *k*-lca vertices for any k≤i, and then removing all shortcuts. As we shall see, this transformation always yields an *i*-lca-relevant and regular DAG; see also Theorem 3.3.

#### Definition 3.2

For an integer i≥1, the *i-regularization* of a DAG *G* is $\textsc {Reg}_i(G):=(G\ominus \overline{\mathfrak {lca}_{i}}(G))^-$. In particular, if *G* is a DAG on *X*, then we define the *regularization* of *G* to be $\textsc {Reg}(G):=\textsc {Reg}_{|X|}(G)$.

**Fig. 3:**
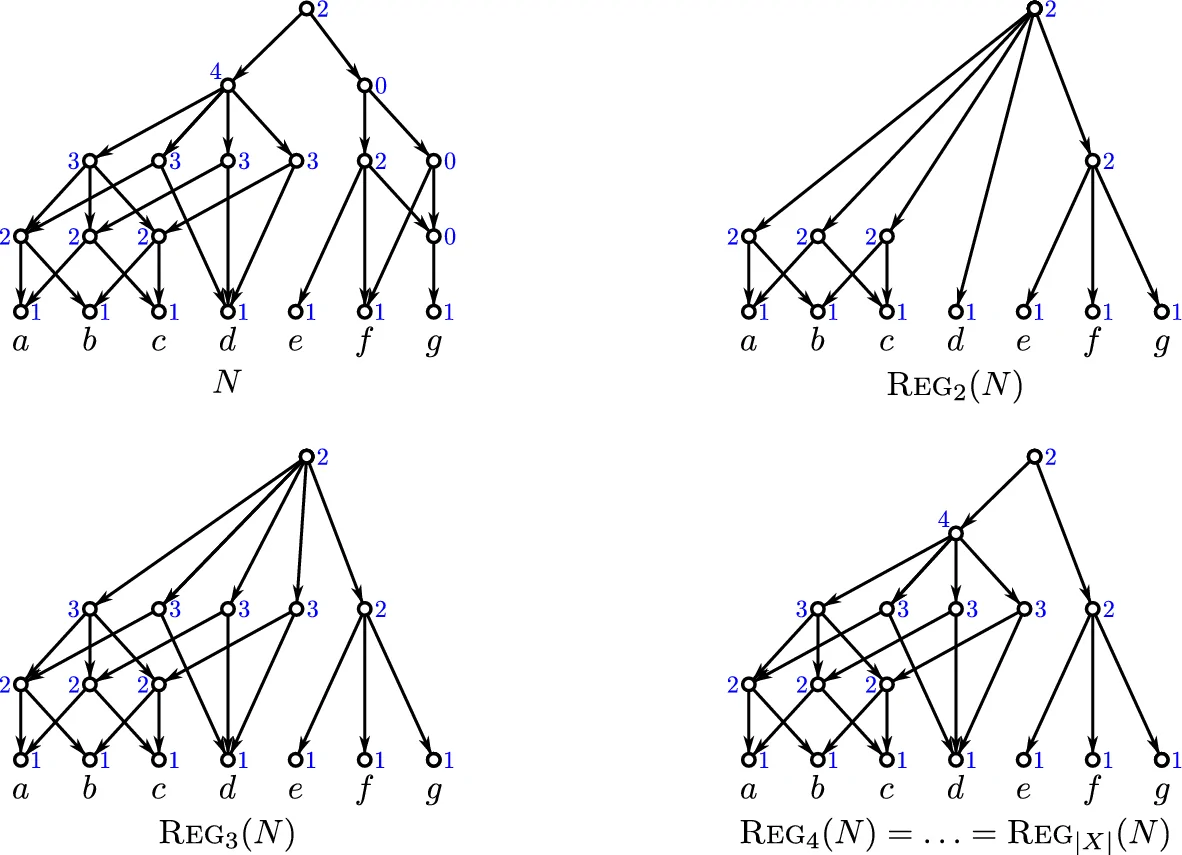
A network *N* on $X=\{a,b,c,d,e,f,g\}$. Numbers *i* next to a vertex *v* denote the smallest integers such that $v\in \mathfrak {lca}_{i}(N)$. If i=0, then the respective vertex is not a *k*-lca vertex for any *k*. In addition several networks $\textsc {Reg}_i(N)$ are shown. Note that $\textsc {Reg}_1(N) = (X,\emptyset )$ just consists of *X* and no arcs

Definition 3.2 is illustrated in Fig. 3. The figure also shows that, for a DAG *G* on *X* and integers j<i, the DAG $\textsc {Reg}_i(G)$ can be viewed as a refinement of $\textsc {Reg}_j(G)$, with the sequence starting at the arc-less DAG $\textsc {Reg}_1(G) = (X,\emptyset )$.

As outlined in the introduction, various approaches to simplify networks have been proposed in recent years. In Lindeberg and Hellmuth (2025), Lindeberg and Hellmuth studied simplifications G⊖W of DAGs *G* in which each vertex v∉W is an *i*-lca vertex for some $i\in \mathscr {I}$, where $\mathscr {I}$ is a set of positive integers. The *i*-regularization of Definition 3.2 is therefore a natural specialization of this construction to $\mathscr {I}=\{1,\ldots ,i\}$, followed by the removal of all shortcuts. In particular, the simplification $\varphi _{\operatorname {lca}}(G)$ introduced in (Lindeberg and Hellmuth, 2025, Sec. 6) coincides with the “full” regularization of *G* as given in Definition 3.2, that is,

$$\textsc {Reg}(G) = (G\ominus \overline{\mathfrak {lca}_{|X|}}(G))^- =\varphi _{\operatorname {lca}}(G).$$

It follows that $\textsc {Reg}(G)$ satisfies three additional “simplification” axioms that are desirable for such transformations, provided that *G* is a DAG satisfying the (CL) property; see (Lindeberg and Hellmuth, 2025, Sec. 6) and Heiss et al. (2025) for further details.

**Fig. 4:**
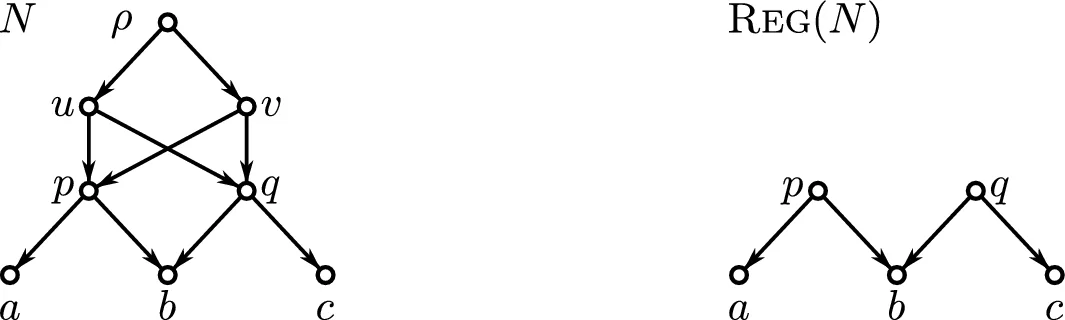
The network *N* has a unique root, while the corresponding DAG $\textsc {Reg}(N)$ has two

**Fig. 5:**
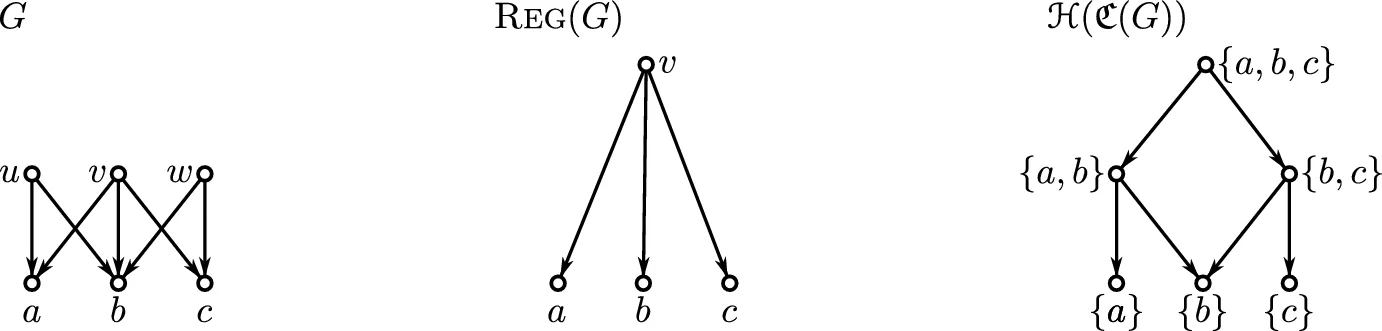
A DAG *G* for which $\mathcal {H}(\mathfrak {C}(G)) \not \simeq \textsc {Reg}(G)$ holds

It is worth noting that, in general, there is no direct relationship between the number of roots of a DAG *G* and the number of roots of $\textsc {Reg}_i(G)$ for i≥1. For example, the network *N* in Fig. 4 loses its unique root under regularization: $\textsc {Reg}(N)$ has two distinct roots. On the other hand, the DAG *G* in Fig. 5 has three roots, while $\textsc {Reg}(G)$ has only one. Thus, even when *G* is a network, $\textsc {Reg}_i(G)$ need not be a network, and conversely, a DAG with several roots may have a regularization that is a network.

Our main motivation for considering $\textsc {Reg}_i(G)$ stems from results established in Lindeberg and Hellmuth (2025), which we restate using our notation.

#### Theorem 3.3

( Lindeberg and Hellmuth (2025)) Let *G* be a DAG on *X* and i≥1 be an integer. Then, $\textsc {Reg}_i(G)$ is an *i*-lca-relevant and regular DAG on *X*. Moreover, *G* is regular if and only if $\textsc {Reg}(G) = G$.

#### Proof

Theorem 5.5 in Lindeberg and Hellmuth (2025) ensures that $G\ominus \overline{\mathfrak {lca}_{i}}(G)$ is *i*-lca-relevant for every DAG *G* and integer i≥1. Since $\textsc {Reg}_i(G)=(G\ominus \overline{\mathfrak {lca}_{i}}(G))^-$ is shortcut-free, Observation 2.13 implies that $\textsc {Reg}_i(G)$ is *i*-lca-relevant. Thus, regularity of $\textsc {Reg}_i(G)$ follows from Theorem 2.12. The second statement is an immediate consequence of the first statement and Theorem 2.12. □

The key point of Theorem 3.3 is that $\textsc {Reg}_i(G)$ is regular, which motivates its name. Consequently, its structure is determined solely by its set of clusters. Although (Lindeberg and Hellmuth 2025) implicitly considers $\textsc {Reg}_i(G)$, it does not provide a description of $\mathfrak {C}(\textsc {Reg}_i(G))$, which we now establish. To this end, we first introduce a new definition.

#### Definition 3.4

Let *G* be a DAG on *X*. The set of *lca-clusters* of *G* is defined to be

$$ \mathfrak {C}_{\operatorname {lca}}(G):=\{C\in \mathfrak {C}(G)\mid \operatorname {lca}_G(C) \text { is well-defined}\}. $$

Furthermore, for each integer $i\in \{1,2,\ldots ,|X|\}$, we define

$$ \mathfrak {C}_{\operatorname {lca}}^{i}(G):=\{C\in \mathfrak {C}_{\operatorname {lca}}(G)\mid \exists A\in \mathscr {P}_{\le i}(X):\, C \text { is the unique inclusion-minimal element in }\mathfrak {C}(G) \text { containing }A\}. $$

Definition 3.4 is illustrated with the help of the following example.

#### Example

In Fig. 2, two networks $N^{\prime }$ and $N^{\prime \prime }$ are shown that both have leaf set $$X=\{a,b,c\}$$. For $N^{\prime }$, we have $$\mathfrak {C}(N') = \{\{a,b,c\}, \{a,b\}, \{a\}, \{b\}, \{c\}\}$$ where $${{\,\mathrm{\texttt{C}}\,}}_{N'}(u)={{\,\mathrm{\texttt{C}}\,}}_{N'}(v)=\{a,b\}$$, i.e., *u* and *v* are not distinguishable from their associated clusters. Neither *u* nor *v* serve as a unique LCA for any subset of leaves. In particular, $${{\,\mathrm{\texttt{C}}\,}}_{N'}(v)={{\,\mathrm{\texttt{C}}\,}}_{N'}(u)=\{a,b\}\notin \mathfrak {C}_{\operatorname {lca}}(N') $$ and we obtain $$\mathfrak {C}_{\operatorname {lca}}(N') = \{\{a,b,c\}, \{a\}, \{b\}, \{c\}\}$$. It is easy to verify that $$\mathfrak {C}_{\operatorname {lca}}^{1}(N') = \{\{a\}, \{b\}, \{c\}\}$$ and $$\mathfrak {C}_{\operatorname {lca}}^{3}(N') = \mathfrak {C}_{\operatorname {lca}}(N')$$. In this example, we even have $$\mathfrak {C}_{\operatorname {lca}}^{2}(N') = \mathfrak {C}_{\operatorname {lca}}(N')$$ since $$\{a,b,c\} \in \mathfrak {C}_{\operatorname {lca}}(N')$$ is the unique inclusion-minimal element in $$\mathfrak {C}(N')$$ that contains for example $$A= \{a,c\} \in \mathscr {P}_{\le i}(X)$$. For $N^{\prime \prime }$, we have $$\mathfrak {C}(N'')=\{A\mid \emptyset \ne A\subseteq X\}=\mathscr {P}_{\le 3}(X)$$ and $$\mathfrak {C}_{\operatorname {lca}}^{i}(N'') =\mathscr {P}_{\le i}(X)$$, $$1\le i\le 3$$.

Note that, by definition, $\mathfrak {C}_{\operatorname {lca}}^{i}(G)\subseteq \mathfrak {C}_{\operatorname {lca}}^{j}(G)$ always holds whenever i≤j. Moreover, there are DAGs *G*, for example phylogenetic trees, for which $\mathfrak {C}_{\operatorname {lca}}(G) = \mathfrak {C}(G)$ holds. We now describe the set of clusters of $\textsc {Reg}_i(G)$.

#### Theorem 3.5

For each DAG *G* on *X* and every $i\in \{1,2,\ldots ,|X|\}$, $\mathfrak {C}(\textsc {Reg}_i(G))=\mathfrak {C}_{\operatorname {lca}}^{i}(G)$. In particular, since $\textsc {Reg}_i(G)$ is regular, $\textsc {Reg}_i(G)\simeq \mathcal {H}(\mathfrak {C}_{\operatorname {lca}}^{i}(G))$.

#### Proof

Let *G* be a DAG on *X*, put ℓ:=|X| and let $i\in \{1,\ldots ,\ell \}$. We first note that the statements in Observation 2.13 are, in particular, valid for $H:=\textsc {Reg}_i(G) = (G\ominus \overline{\mathfrak {lca}_{i}}(G))^-$ and that $V(H)=\mathfrak {lca}_{i}(G)$. Hence, to prove the theorem, we show that $\mathfrak {C}(H)=\mathfrak {C}_{\operatorname {lca}}^{i}(G)$.

First, suppose $C\in \mathfrak {C}(H)$. By definition, there exists some v∈V(H) such that ${{\,\mathrm{\texttt{C}}\,}}_{H}(v)=C$. Recall that, from Observation 2.13, ${{\,\mathrm{\texttt{C}}\,}}_{H}(v)={{\,\mathrm{\texttt{C}}\,}}_{G}(v)$. Moreover, since $v\in \mathfrak {lca}_{i}(G)=V(H)$ there is some $A\in \mathscr {P}_{\le i}(X)$ such that $v=\operatorname {lca}_G(A)$. The latter implies that ${{\,\mathrm{\texttt{C}}\,}}_G(\operatorname {lca}_G(A)) = {{\,\mathrm{\texttt{C}}\,}}_G(v)=C$. Moreover, since $\operatorname {lca}_G(A)$ is well-defined, we can apply Lemma 2.9 to conclude that $\operatorname {lca}_G({{\,\mathrm{\texttt{C}}\,}}_G(\operatorname {lca}_G(A)))=\operatorname {lca}_G(C)$ is well-defined (and equals *v*). In other words, $C\in \mathfrak {C}_{\operatorname {lca}}(G)$. Moreover, Lemma 2.9 also ensures that $C={{\,\mathrm{\texttt{C}}\,}}_G(\operatorname {lca}_G(A))$ is the unique inclusion-minimal cluster in $\mathfrak {C}(G)$ that contains *A*. We have thus shown that $C\in \mathfrak {C}_{\operatorname {lca}}^i(G)$. In other words, $\mathfrak {C}(H)\subseteq \mathfrak {C}_{\operatorname {lca}}^{i}(G)$.

To see that $$\mathfrak {C}_{\operatorname {lca}}^{i}(G)\subseteq \mathfrak {C}(H)$$ holds, consider any cluster $$C\in \mathfrak {C}_{\operatorname {lca}}^{i}(G)$$. One easily verifies that there exists some v∈V(G) such that $${{\,\mathrm{\texttt{C}}\,}}_G(v)=C$$ and $$v=\operatorname {lca}_G(C)$$, see Lemma 2.9. We now claim that v∈V(H). Since, by assumption, $$C\in \mathfrak {C}_{\operatorname {lca}}^{i}(G)$$, there exists, by definition, some $$A\in \mathscr {P}_{\le i}(X)$$ such that *C* is the unique inclusion-minimal cluster in $$\mathfrak {C}(G)$$ containing *A*. Since A⊆C and $$v=\operatorname {lca}_G(C)$$, *v* is a common ancestor of *A* and $$\operatorname {LCA}_G(A)\ne \emptyset $$. Consider any $$v'\in \operatorname {LCA}_G(A)$$. Since $$A\subseteq {{\,\mathrm{\texttt{C}}\,}}_G(v')$$ and *C* is the unique inclusion-minimal cluster in $$\mathfrak {C}(G)$$ containing *A*, we must have $$C\subseteq {{\,\mathrm{\texttt{C}}\,}}_G(v')$$. Hence, $v^{\prime }$ is a common ancestor *C*. The latter together with the fact that $$v=\operatorname {lca}_G(C)$$ is the unique least common ancestor of *C* implies that $$v=\operatorname {lca}_G(C)\preceq _G v'$$ must hold. As the latter holds for every $$v'\in \operatorname {LCA}_G(A)$$, we can conclude that $$\operatorname {LCA}_G(A)=\{v\}$$, i.e., that $$v=\operatorname {lca}_G(A)$$. Since $$A\in \mathscr {P}_{\le i}(X)$$, it follows that $$v\in \mathfrak {lca}_{i}(G)=V(H)$$, as claimed.

Observation 2.13 then implies that $C={{\,\mathrm{\texttt{C}}\,}}_G(v)={{\,\mathrm{\texttt{C}}\,}}_{H}(v)\in \mathfrak {C}(H)$ i.e. $\mathfrak {C}_{\operatorname {lca}}^{i}(G)\subseteq \mathfrak {C}(H)$. Finally, to complete the proof, observe that Theorem 3.3 implies that $\textsc {Reg}_i(G)$ is regular. By definition of regular DAGs, $\textsc {Reg}_i(G)\simeq \mathcal {H}(\mathfrak {C}_{\operatorname {lca}}^{i}(G))$. □

Theorem 3.5 provides a characterization of the structure of $\textsc {Reg}_i(G)$ in terms of certain clusters of *G*. In the rest of this section, we shall pinpoint consequences of this result and explore further properties of the *i*-regularization operator.

A natural starting point is to consider the regularization of the archetypal phylogenetic structures, namely trees. One easily verifies that, in a tree, a vertex *v* is a *k*-lca vertex for some *k* if and only if *v* does not have out-degree one. Hence, $\overline{\mathfrak {lca}_{i}}(T)$ is precisely the set of vertices of out-degree one in any tree *T* and for all i≥2. Moreover, there is a well-known bijective correspondence between phylogenetic trees on *X* and hierarchies on *X*, see e.g. (Semple and Steel, 2003, Thm. 3.5.2), Thus, Theorem 3.5 immediately characterizes those graphs *G* for which $\textsc {Reg}_i(G)$ is a tree. We collect these observations in the following statement.

#### Observation 3.6

For all i≥2, the *i*-regularization $$\textsc {Reg}_i(T)$$ of a tree *T* on *X* coincides with $T^{\prime }$, where $T^{\prime }$ is the tree obtained from *T* by suppressing all vertices of out-degree one. In particular, $$\textsc {Reg}_i(T)=T$$ for all phylogenetic trees *T* and all i≥2. Moreover, for any DAG *G* and i≥2, the graph $$\textsc {Reg}_i(G)$$ is a tree if and only if $$\mathfrak {C}_{\operatorname {lca}}^{i}(G)$$ is a hierarchy.

Another straightforward observation is that LCAs are unaffected by the presence or absence of shortcuts. Consequently, adding or removing shortcuts in *G* does not affect $\textsc {Reg}_i(G)$. This can be formalized as follows.

#### Lemma 3.7

For every DAG *G* and integer i≥1, $\textsc {Reg}_i(G)=\textsc {Reg}_i(G^-)$.

#### Proof

Recall that, by definition, $V(G)=V(G^-)$ and that Lemma 2.1 states that $u\preceq _G v$ if and only if $u\preceq _{G^-}v$ for all u,v∈V(G). It is now straightforward to verify that, for every non-empty A⊆X, $\operatorname {LCA}_G(A)=\operatorname {LCA}_{G^-}(A)$ and, therefore, $\mathfrak {lca}_{i}(G)=\mathfrak {lca}_{i}(G^-)$. This together with Lemma 2.7 implies that $V(\textsc {Reg}_i(G))=\mathfrak {lca}_{i}(G)=V(\textsc {Reg}_i(G^-))$. Since $\textsc {Reg}_i(G)$ and $\textsc {Reg}_i(G^-)$ are shortcut-free the latter, taken together with the last statement of Lemma 2.1, implies that $\textsc {Reg}_i(G)=\textsc {Reg}_i(G^-)$. □

Note that $\mathcal {H}(\mathfrak {C}(G))$ and $\textsc {Reg}(G)$ may differ; see Fig. 5. In this example, for the DAG *G* we have $v=\operatorname {lca}_G(a,c)$, while neither *u* nor *w* is the unique LCA of any subset of the leaves. As a result, $\textsc {Reg}(G)$ is just a “star tree”. In contrast, $\mathcal {H}(\mathfrak {C}(G))$ not only differs substantially from *G* but also from $\textsc {Reg}(G)$. As the next results shows, $\mathcal {H}(\mathfrak {C}(G))$ and $\textsc {Reg}(G)$ are isomorphic precisely if $\mathfrak {C}(G)=\mathfrak {C}_{\operatorname {lca}}(G)$.

#### Theorem 3.8

For every DAG *G*, $\textsc {Reg}(G)\simeq \mathcal {H}(\mathfrak {C}_{\operatorname {lca}}(G))$. Moreover, $\textsc {Reg}(G)\simeq \mathcal {H}(\mathfrak {C}(G))$ if and only if *G* satisfies property (CL).

#### Proof

Let *G* be a DAG on *X* and put ℓ=|X|. Theorem 3.5 states that $\mathfrak {C}(\textsc {Reg}(G))=\mathfrak {C}(\textsc {Reg}_\ell (G))=\mathfrak {C}_{\operatorname {lca}}^{\ell }(G)$. By definition and Lemma 2.9, $\mathfrak {C}_{\operatorname {lca}}^{\ell }(G)=\mathfrak {C}_{\operatorname {lca}}(G)$ holds. Since, by Theorem 3.3, $\textsc {Reg}(G)$ is regular, the latter two arguments ensure that $\textsc {Reg}(G)\simeq \mathcal {H}(\mathfrak {C}_{\operatorname {lca}}(G))$ for all DAGs *G*. The second statement now follows immediately from the fact that, by definition, *G* satisfies property (CL) if and only if $\operatorname {lca}_G({{\,\mathrm{\texttt{C}}\,}}_G(v))$ is well-defined for all v∈V(G), which is, if and only if $\mathfrak {C}(G)=\mathfrak {C}_{\operatorname {lca}}(G)$. □

We close this section by discussing some computational aspects of *i*-regularization. As a starting point, note that the property of being 2-lca-relevant can be tested in polynomial time. More generally, for every fixed constant integer *k*, testing whether a DAG is *k*-lca-relevant can be done in polynomial time; see (Lindeberg and Hellmuth, 2025, Obs. 7.6).. Moreover, (Lindeberg and Hellmuth, 2025, Cor. 3.12) gives a polynomial-time algorithm for testing whether a given DAG is lca-relevant. In contrast, testing whether a DAG *G* is *k*-lca-relevant when *k* is part of the input is NP-complete (Lindeberg and Hellmuth, 2025, Thm. 7.5). We now extend these results to the computation of $\textsc {Reg}_i(G)$ for certain values of *i*.

#### Proposition 3.9

Given a DAG G=(V,E) on *X*, the DAG $\textsc {Reg}(G)$ can be constructed in polynomial time. Moreover, if *i* is a fixed constant, then $\textsc {Reg}_i(G)$ can be constructed in polynomial time in |*V*|.

#### Proof

Let G=(V,E) be a DAG on *X*. By definition, $\textsc {Reg}(G) = (G\ominus \overline{\mathfrak {lca}_{|X|}}(G))^-$ and $\overline{\mathfrak {lca}_{|X|}}(G) = \{v\in V \mid v \text { is not a } k\text {-lca vertex for any } k\le |X|\}$. Together with Lemma 2.9, this implies that $\overline{\mathfrak {lca}_{|X|}}(G) = \{v\in V \mid v\ne \operatorname {lca}_G({{\,\mathrm{\texttt{C}}\,}}_G(v))\}$. For each vertex v∈V, the set $\operatorname {LCA}_G({{\,\mathrm{\texttt{C}}\,}}_G(v))$ can be computed in polynomial time using the algorithm from Lindeberg and Hellmuth (2025). Hence, we can determine in polynomial time whether $v=\operatorname {lca}_G({{\,\mathrm{\texttt{C}}\,}}_G(v))$, and thus construct $\overline{\mathfrak {lca}_{|X|}}(G)$ in polynomial time. Finally, $\textsc {Reg}(G)=(G\ominus \overline{\mathfrak {lca}_{|X|}}(G))^-$ can be computed in $O(|V|^2)$ time; see (Lindeberg and Hellmuth, 2025, Section 5) for further details.

Now let *i* be a fixed constant. We can determine $\overline{\mathfrak {lca}_{i}}(G)$ by computing $\operatorname {LCA}_G(A)$ for all subsets A⊆X with |A|≤i. There are $O(|X|^i)$ such subsets, and each corresponding LCA set can be computed in polynomial time using the algorithm from Lindeberg and Hellmuth (2025). Since *i* is fixed, this yields a polynomial-time algorithm for constructing $\overline{\mathfrak {lca}_{i}}(G)$. As above, $\textsc {Reg}_i(G)=(G\ominus \overline{\mathfrak {lca}_{i}}(G))^-$ can then be computed in polynomial time. □

### Regularizing Normal Networks

In this section, we investigate how LCA-based regularization acts on normal networks and, in particular, clarify the relationships between normality, strong normality, and lca-relevance. We show that regularization preserves normality and characterize precisely when $\textsc {Reg}(N)=N$; see Theorem 3.14. These structural results also provide important ingredients for Sect. 4, where we compare regularization with normalization within the common ⊖-framework.

**Fig. 6:**
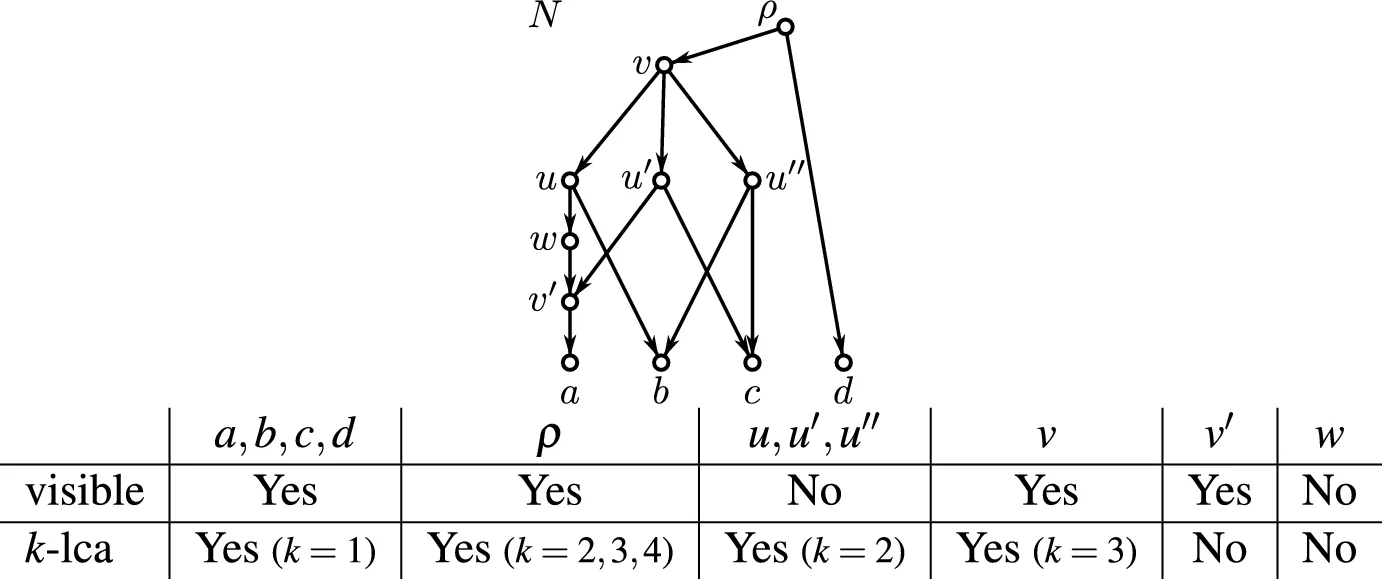
A network *N* on $\{a,b,c,d\}$. The table specifies which of the vertices of *N* are (non-)visible or (non-)*k*-lca vertices

We note that the property of a vertex being visible and being a *k*-lca vertex are two unrelated properties; neither of them implies the other. In fact, there are networks *N* containing vertices of every possible combination of (non-)visible and (non-)*k*-lca vertices, see *N* and the table in Fig. 6. Nevertheless, as the next result shows, every strongly normal network is *k*-lca-relevant for k≥2. Thus, all vertices are *k*-lca vertices and, by Theorem 2.4, at the same time visible. This result has, in parts, been proven by Willson (Willson, 2010, Thm 3.9), albeit in somewhat different setting and using alternative notation.

#### Lemma 3.10

Every strongly normal DAG on *X* is *k*-lca-relevant for all $k\in \{2,\dots ,|X|\}$.

#### Proof

Since the proof is straightforward, we have placed it in Appendix A. □

We now provide some characterizations of strongly normal networks.

#### Proposition 3.11

Let *N* be a network. Then, the following four statements are equivalent.

1. *N* is strongly normal.
2. *N* is normal and 2-lca-relevant.
3. *N* is normal and lca-relevant.
4. *N* is normal, phylogenetic and does not contain hybrids having only one child.

#### Proof

Lemma 3.10 shows that Condition (1) implies (2), and the implication from (2) to (3) is immediate by definition. If *N* is normal and lca-relevant, then the contraposition of Lemma 2.10 implies that no vertex of *N* has out-degree one. Hence, Condition (3) implies (1). The equivalence between (1) and (4) follows from the fact that, in a phylogenetic network, only hybrid vertices can have out-degree one. □

We now show that, for a normal DAG *G*, normality is preserved under the ⊖-operator when applied to vertices of out-degree one, and that these vertices are precisely those contained in $\overline{\mathfrak {lca}_{i}}(G)$.

#### Proposition 3.12

Let *G* be a normal DAG on *X* and $W\subseteq U:=\{v\in V(G)\mid {{\,\textrm{outdeg}\,}}_G(v)=1\}$. Then, G⊖W is a normal DAG on *X* and G⊖U is strongly normal. In particular, $\overline{\mathfrak {lca}_{i}}(G)=U$ for all integers i≥2.

#### Proof

Since the proof is straightforward but technical, we have placed it in Appendix A. □

**Fig. 7:**
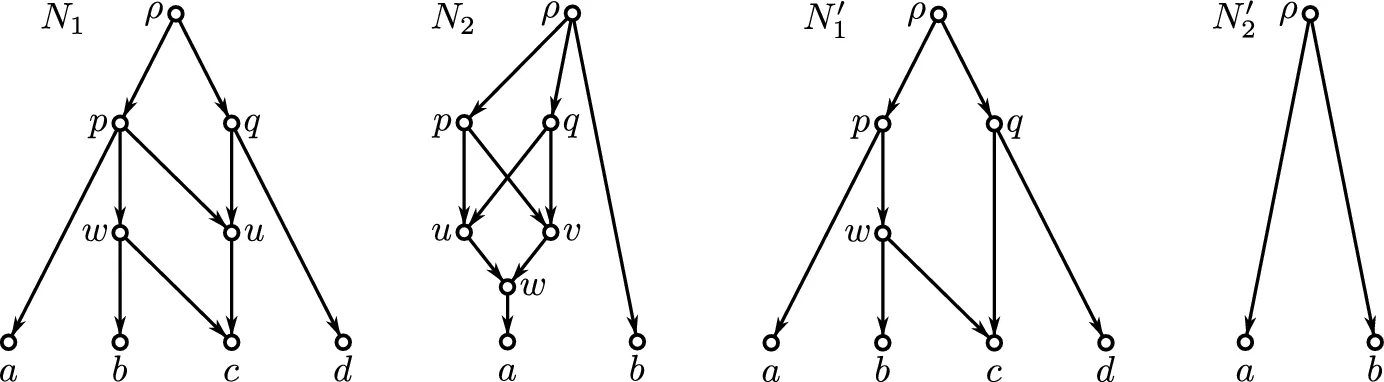
Two networks $N_1$ and $N_2$ for which the regularization and normalization coincide, i.e., $\textsc {Reg}(N_1)=N_1'=\textsc {Norm}(N_1)$ and $\textsc {Reg}(N_2)=N_2'=\textsc {Norm}(N_2)$

Note that normal networks are not the only type of DAGs for which $\overline{\mathfrak {lca}_{i}}(N)=\{v\in V(N)\mid {{\,\textrm{outdeg}\,}}_N(v)=1\}$. For example, consider the network $N_1$ in Fig. 7, for which $\overline{\mathfrak {lca}_{i}}(N_1)=\{u\}$ for all i≥2, i.e., $\overline{\mathfrak {lca}_{i}}(N_1)$ contains precisely the only vertex with out-degree one. Here, the vertex *u* has no tree-child and thus $N_1$ is not normal. Nevertheless, $N_1$ is phylogenetic and shortcut-free.

A straightforward consequence of Proposition 3.12 is the following. For a normal DAG *G*, the graph $G\ominus \overline{\mathfrak {lca}_{2}}(G)$ is already shortcut-free. Moreover, $\overline{\mathfrak {lca}_{i}}(G)=\overline{\mathfrak {lca}_{2}}(G)$ for all i≥2, and hence $\textsc {Reg}_i(G)$ is obtained simply by removing the vertices of out-degree one. Hence, we obtain

#### Corollary 3.13

For every normal DAG *G* it holds that $\textsc {Reg}(G) = \textsc {Reg}_i(G) = G\ominus \overline{\mathfrak {lca}_{2}}(G)$ for all integers i≥2, and, in particular, $V(\textsc {Reg}(G))=\mathfrak {lca}_{2}(G)$.

Recall that $\textsc {Reg}(N)$ may have multiple roots, even though *N* itself is a network (c.f. Fig. 4). Nevertheless, the latter cannot happen if *N* is a normal network as we now show in the main result of this section.

#### Theorem 3.14

Let *N* be a normal network on *X*. Then, $\textsc {Reg}(N)$ is a normal phylogenetic network on *X*. In particular, the following statements are equivalent.

1. $$\textsc {Reg}(N) = N$$.
2. *N* is regular.
3. *N* is strongly normal.

Moreover, if |X|≥2, then Statements (1), (2), (3) and

(4) $$\textsc {Reg}_i(N) = N$$ for all $$i \in \{2,\dots , |X|\}$$

are equivalent.

#### Proof

Let *N* be a normal network on *X* and let ρ be its unique root. By Corollary 3.13 and Proposition 3.12 we have that $\textsc {Reg}(N)=N\ominus \overline{\mathfrak {lca}_{2}}(N)$, where $\overline{\mathfrak {lca}_{2}}(N)=\{v\in V(N)\mid {{\,\textrm{outdeg}\,}}_N(v)=1\}$. This together with Proposition 3.12 implies that $\textsc {Reg}(N)$ is a normal DAG on *X*. We show that $\textsc {Reg}(N)$ is a network, i.e, that $\textsc {Reg}(N)$ has a unique root.

If $\rho \in V(\textsc {Reg}(N))$, then $v\preceq _N \rho$ for all v∈V(N). Observation 2.13 therefore implies $v \preceq _{\textsc {Reg}(N)} \rho$, for all $v\in V(\textsc {Reg}(N))$ and hence, ρ remains the unique root of $\textsc {Reg}(N)$. Now suppose that $\rho \not \in V(\textsc {Reg}(N))$, that is, ${{\,\textrm{outdeg}\,}}_N(\rho ) = 1$. Starting at ρ, follow the unique outgoing arc until reaching the first vertex *v* with ${{\,\textrm{outdeg}\,}}_N(v)\ne 1$; such a vertex exists and may be a leaf. Then *v* is the unique $\preceq _N$-maximal vertex of *N* whose out-degree is distinct from one. By Observation 2.13, $u\preceq _{\textsc {Reg}(N)}v$ for every $u\in V(\textsc {Reg}(N))$. Thus, *v* is the unique root of $\textsc {Reg}(N)$. In summary, $\textsc {Reg}(N)$ is a normal *network* on *X*. Moreover, Theorem 3.3 implies that $\textsc {Reg}(N)$ is regular and thus, by (Lindeberg and Hellmuth, 2025, Thm 4.6), also phylogenetic.

We now prove the remaining statements in the theorem. By Theorem 3.3, Statements (1) and (2) are equivalent. Suppose that (1) holds. Since *N* is shortcut-free, $N^- = N =\textsc {Reg}(N)$. This together with Theorem 3.3 implies that *N* is lca-relevant. Since *N* is normal, Proposition 3.11 implies that *N* is strongly normal. Hence, (1) implies (3). Suppose now that (3) holds.

Suppose first that |X|=1 and let $X=\{x\}$. If *N* has more than one vertex, then its root ρ must have at least two distinct children *u* and *v*, since *N* is strongly normal. Since *N* is normal, Theorem 2.4 implies that both *u* and *v* are visible. Since $X=\{x\}$, visibility of *u* means that every ρx-path passes through *u*, and visibility of *v* means that every ρx-path passes through *v*. Hence, any ρx-path starting with the arc $\rho \rightarrow u$ must later pass through *v*, so $v\prec _N u$. Similarly, any ρx-path starting with $\rho \rightarrow v$ must later pass through *u*, so $u\prec _N v$, contradicting acyclicity. Hence, *N* consists of its single leaf and thus $\textsc {Reg}(N)=N$. Therefore, (3) implies (1) in case |X|=1. In other words, Statements (1), (2) and (3) are equivalent in case |X|=1.

Suppose now that |X|≥2. Lemma 3.10 implies that *N* is *i*-lca-relevant for all $i \in \{2,\dots , |X|\}$. Thus, $\overline{\mathfrak {lca}_{i}}(N)=\emptyset$ and since *N* is shortcut-free, we obtain $N = N^-=(N\ominus \overline{\mathfrak {lca}_{i}}(N))^-=\textsc {Reg}_{i}(N)$ for all $i \in \{2,\dots , |X|\}$. Hence, (3) implies (4). Finally, since $\textsc {Reg}(N) = \textsc {Reg}_{|X|}(N)$ and |X|≥2, Statement (4) immediately implies (1). Therefore, Statements (1), (2), (3) and (4) are equivalent in case |X|≥2. □

Note, in particular, that Statements (2) and (4) of Theorem 3.14 are not necessarily equivalent if *N* is not normal. For example, the network $N^{\prime \prime }$ in Fig. 2 is regular, but $$\textsc {Reg}_2(N'')=N''\ominus \rho \ne N''$$.

## Normalizing versus Regularizing

In Francis et al. (2021) the concept of *normalization* was introduced, that is, the transformation of a given network *N* into a normal version $\textsc {Norm}(N)$ of the network that aims to retain the underlying evolution information whilst producing a network that has the desirable features of a normal network. Since clustering systems naturally arise in network inference, $\textsc {Reg}(N)$ – by virtue of being regular – also encodes useful phylogenetic signals through leaf-based information, since every vertex is the unique LCA of some subset of leaves (cf. Theorem 3.3). It is therefore natural to investigate how $\textsc {Reg}(N)$ and $\textsc {Norm}(N)$ are related, as this could be useful information in practice when using these techniques.

One of the main goals of this section is to characterize those networks for which $\textsc {Reg}(N)= \textsc {Norm}(N)$ holds. In particular, we show that $\textsc {Norm}(N)$ can be fully expressed as $(N\ominus W)^-$ for a particular subset W⊆V(N) and thus, conceptually, falls within the same paradigm as $\textsc {Reg}(N)$. Before providing the definition of $\textsc {Norm}(N)$, we emphasize that (implicitly), all networks in Francis et al. (2021) are separated, i.e., all hybrids *v* in *N* have ${{\,\textrm{outdeg}\,}}_N(v)=1$. Moreover, no vertex *v* of the networks used in Francis et al. (2021) satisfies ${{\,\textrm{indeg}\,}}(v)={{\,\textrm{outdeg}\,}}(v)=1$. Hence, they are phylogenetic “up to” possibly the root having out-degree 1. We thus slightly generalize the definition of $\textsc {Norm}(N)$ to arbitrary networks *N*.

### Definition 4.1

*(Francis et al.,* 2021,*Sec. 2)* Let *N* be a network. The directed graphs $\textsc {Cov}(N)$ and $\textsc {Norm}(N)$ are constructed as follows.

1. Let $G = (\mathfrak {vis}(N),\,A)$ be the DAG whose arc set *A* comprises all arcs u→v for which $u,v\in \mathfrak {vis}(N)$ and $v\prec _N u$ holds
2. Put $$\textsc {Cov}(N) = G^-$$.
3. $\textsc {Norm}(N)$ is obtained from $\textsc {Cov}(N)$ by suppressing all vertices *v* of $\textsc {Cov}(N)$ for which ${{\,\textrm{indeg}\,}}_{\textsc {Cov}(N)}(v)={{\,\textrm{outdeg}\,}}_{\textsc {Cov}(N)}(v)=1$.

**Fig. 8:**
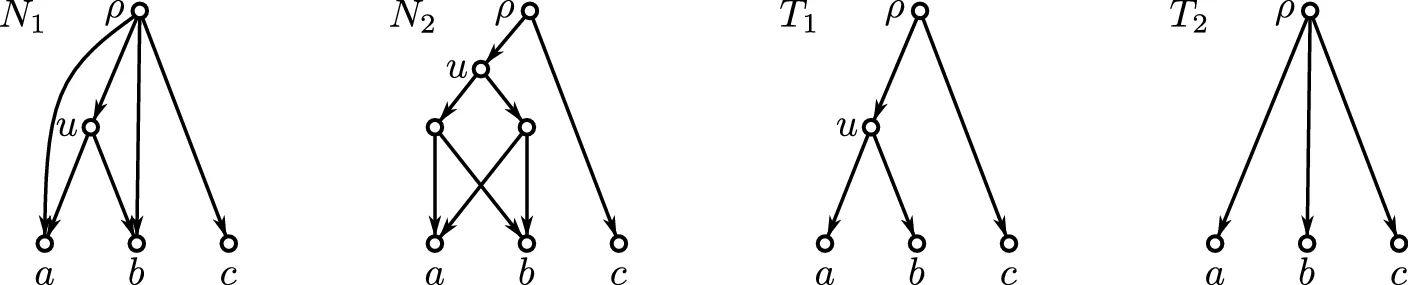
Two networks $N_1$, $N_2$ and two trees $T_1$, $T_2$ on $\{a,b,c\}$, for which $T_1 =\textsc {Reg}(N_1) = \textsc {Norm}(N_2)$ and $T_2=\textsc {Norm}(N_1)=\textsc {Reg}(N_2)$

There are many networks *N* for which the normalization $\textsc {Norm}(N)$ and the regularization $\textsc {Reg}(N)$ of *N* coincide. For example, considering the networks shown in Fig. 7, we have both $N_1'=\textsc {Norm}(N_1)=\textsc {Reg}(N_1)$ and $N_2'=\textsc {Norm}(N_2)=\textsc {Reg}(N_2)$. However, this is not always the case. For example, see Fig. 8 where $\textsc {Norm}(N_1)=\textsc {Reg}(N_2)=T_2\ne T_1 =\textsc {Reg}(N_1) = \textsc {Norm}(N_2)$. In particular, there are lca-vertices that are not visible (e.g. vertex *u* in $N_1$) and there are visible vertices that are not lca-vertices (e.g. the vertex *u* in $N_2$). In other words, $\textsc {Reg}(N)$ and $\textsc {Norm}(N)$ may identify different vertices as “omittable”. A technical difficulty in comparing regularization and normalization is that the operator $\textsc {Reg}$ is invariant under the addition or removal of shortcuts. More precisely, $\textsc {Reg}(N^-)=\textsc {Reg}(N)$ always holds; cf. Lemma 3.7(2). In contrast, the normalization operator $\textsc {Norm}$ does not share this invariance property.

To illustrate this fact, consider the networks shown in Fig. 8. The network $N_1$ differs from $T_1$ only by the presence of the two shortcuts $\rho \rightarrow a$ and $\rho \rightarrow b$. Although $\textsc {Reg}(N_1^-)=\textsc {Reg}(N_1)=T_1$, we have $\textsc {Norm}(N_1)=T_2 \ne T_1 = \textsc {Norm}(N_1^-)$. Thus, in general, $\textsc {Norm}(N) \ne \textsc {Norm}(N^-)$. Nevertheless, we show that normalization can be characterized using the ⊖-operator together with the removal of shortcuts. To this end, we start with establishing the connection between the graph $\textsc {Cov}(N)$ in Definition 4.1 and the ⊖-operator combined with shortcut removal.

### Lemma 4.2

For all networks *N* on *X*, $\textsc {Cov}(N)=(N\ominus \overline{\mathfrak {vis}}(N))^-$ is a normal network on *X*.

### Proof

Since the proof is straightforward, we have placed it in Appendix A. □

To express $\textsc {Norm}(N)$ in terms of the ⊖-operator, we need to determine the set *W* for which $\textsc {Norm}(N)=(N\ominus W)^-$ holds. To this end, we define the following set

$$ \mathfrak {dvis}(N) :=\{v \in \mathfrak {vis}(N) \mid {{\,\textrm{indeg}\,}}_{\textsc {Cov}(N)}(v) = {{\,\textrm{outdeg}\,}}_{\textsc {Cov}(N)}(v) = 1\}. $$

The vertices in $\mathfrak {dvis}(N)$ are called *dispensably visible* (or $\operatorname {d-vis}$) vertices. By definition, $\textsc {Norm}(N) = \textsc {Cov}(N)$ if and only if $\textsc {Cov}(N)$ contains no vertices *u* with ${{\,\textrm{indeg}\,}}_{\textsc {Cov}(N)}(u) = {{\,\textrm{outdeg}\,}}_{\textsc {Cov}(N)}(u) = 1$, that is, if and only if *N* contains no $\operatorname {d-vis}$ vertices. Importantly, the $\operatorname {d-vis}$ vertices in $\mathfrak {vis}(N)$ are determined in terms of their properties in $\textsc {Cov}(N)$. Nevertheless, as the next result shows $\operatorname {d-vis}$ vertices of *N* can be identified solely based on properties they satisfy in *N* itself.

### Lemma 4.3

Let *N* be a network on *X*. We define the following two sets for all vertices $v\in \mathfrak {vis}(N)$:

$$\begin{aligned} P_{\operatorname {vis}}(v)&:=\{u\in \mathfrak {vis}(N)\setminus \{v\}\mid \text {there is some } uv\text {-path } P \text { in } N \text { s.t. } V(P)\cap \mathfrak {vis}(N)=\{u,v\}\} \\ C_{\operatorname {vis}}(v)&:=\{u\in \mathfrak {vis}(N)\setminus \{v\}\mid \text {there is some } vu\text {-path } P \text { in } N \text { s.t. } V(P)\cap \mathfrak {vis}(N)=\{u,v\}\} \end{aligned}$$

Then, for all $v\in \mathfrak {vis}(N)$, the following two statements are equivalent.

$$v\in \mathfrak {dvis}(N)$$.
(a) $P_{\operatorname {vis}}(v)\ne \emptyset$ and $P_{\operatorname {vis}}(v)$ contains a unique $\preceq _N$-minimal vertex and (b) $C_{\operatorname {vis}}(v)\ne \emptyset$ and $C_{\operatorname {vis}}(v)$ contains a unique $\preceq _N$-maximal vertex.

Moreover, if $v\in \mathfrak {dvis}(N)$, then $v\notin \operatorname {LCA}_N(A)$ for all non-empty A⊆X. In particular, $\mathfrak {dvis}(N)\subseteq \overline{\mathfrak {lca}_{i}}(N)$ for all integers i≥2.

### Proof

Let *N*, $P_{\operatorname {vis}}(v)$ and $C_{\operatorname {vis}}(v)$ be defined as in the lemma’s statement for some $v\in \mathfrak {vis}(N)$. We recall that Lemma 4.2 and Observation 2.13 together implies that

1 $$\begin{aligned} v\preceq _{\textsc {Cov}(N)} u \iff v\preceq _N u,\quad \text { for all }u,v\in \mathfrak {vis}(N)=V(\textsc {Cov}(N)). \end{aligned}$$

First assume that *v* is $\operatorname {d-vis}$ and thus, that ${{\,\textrm{indeg}\,}}_{\textsc {Cov}(N)}(v)={{\,\textrm{outdeg}\,}}_{\textsc {Cov}(N)}(v)=1$. Let *c* denote the unique child of *v* in $\textsc {Cov}(N)$. Hence, $v,c\in \mathfrak {vis}(N)$ and there is no vertex *u* of $\textsc {Cov}(N)$ such that $c\prec _{\textsc {Cov}(N)}u\prec _{\textsc {Cov}(N)} v$. This together with the definition of $\textsc {Cov}(N)$ implies that $c\prec _N v$ and that there is no $u \in \mathfrak {vis}(N)$ such that $c\prec _{N}u\prec _{N} v$. The latter two arguments imply that every path P=v⇝c in *N* must satisfy $V(P)\cap \mathfrak {vis}(N)=\{v,c\}$ and, therefore, $c\in C_{\operatorname {vis}}(v)$. Let $w\in C_{\operatorname {vis}}(v)$ be a vertex such that w≠c. By definition of $C_{\operatorname {vis}}(v)$, it holds that v≠w and there is a *vw*-path in *N* and thus, $w\prec _N v$ and, furthermore, $w\in \mathfrak {vis}(N) = V(\textsc {Cov}(N))$ which together with Eq. (1) implies that $w\prec _{\textsc {Cov}(N)} v$. Since *c* is the unique child of *v* in $\textsc {Cov}(N)$ and w≠c, we therefore have $w\prec _{\textsc {Cov}(N)} c$ which, again, is equivalent to $w\prec _N c$. In other words, *c* must be the unique $\preceq _N$-maximal vertex in the set $C_{\operatorname {vis}}(v)$. With analogous arguments, one shows that the unique parent *p* of *v* in $\textsc {Cov}(N)$ is the unique $\preceq _N$-minimal element of $P_{\operatorname {vis}}(v)$.

Assume, for the converse, that $P_{\operatorname {vis}}(v)$ contains a unique $\preceq _N$-minimal vertex *p* and that $C_{\operatorname {vis}}(v)$ contains a unique $\preceq _N$-maximal vertex *c*. We first show that if an arc v→u of $\textsc {Cov}(N)$ exist, then $u\in C_{\operatorname {vis}}(v)$. To this end, note first that if v→u is an arc of $\textsc {Cov}(N)$, then, by definition, $u\prec _N v$. Let *P* denote any path from *v* to *u* in *N*. If there would exist a visible vertex $w\in V(P)\setminus \{u,v\}$, then $u\prec _N w\prec _N v$ and Eq. (1) would imply that $u\prec _{\textsc {Cov}(N)} w\prec _{\textsc {Cov}(N)} v$; but that would make the arc v→u a shortcut of $\textsc {Cov}(N)$, contradicting $\textsc {Cov}(N)$ being shortcut-free. Hence $V(P)\cap \mathfrak {vis}(N)=\{u,v\}$ and, therefore, $u\in C_{\operatorname {vis}}(v)$. Thus, any arc v→u that potentially exists in $\textsc {Cov}(N)$ must satisfy $u\in C_{\operatorname {vis}}(v)$. Now, by definition, each $u\in C_{\operatorname {vis}}(v)$ satisfies $u\prec _N v$. Hence, for each $u\in C_{\operatorname {vis}}(v)$, the arc v→u is initially added to the DAG *G* as in Definition 4.1(1) from which $\textsc {Cov}(N) = G^-$ is obtained from. Since *c* is the unique $\preceq _N$-maximal vertex in $C_{\operatorname {vis}}(v)$ it follows that any other vertex w≠c in $C_{\operatorname {vis}}(v)$ (if there is one), must satisfy $w\prec _N c$. By the latter arguments, the arcs v→c, c→w and v→w exists in *G*. However, since the arcs v→w is a shortcut in *G*, it will thus not remain in $\textsc {Cov}(N)=G^-$. As this holds for all vertices $w\in C_{\operatorname {vis}}(v){\setminus } \{c\}$ it follows that only the arc v→c exists in $\textsc {Cov}(N)$ and therefore, that *c* is the unique child of *v* in $\textsc {Cov}(N)$. In summary, ${{\,\textrm{outdeg}\,}}_{\textsc {Cov}(N)} = 1$. Using similar arguments, one can show that the unique $\preceq _N$-minimal vertex *p* in $P_{\operatorname {vis}}(v)$ is the unique parent of *v* in $\textsc {Cov}(N)$ and thus, that ${{\,\textrm{indeg}\,}}_{\textsc {Cov}(N)} = 1$. By definition, $v\in \mathfrak {dvis}(N)$. Therefore Statement (1) and (2) are equivalent.

Now consider any $v\in \mathfrak {dvis}(N)$. Since ${{\,\textrm{outdeg}\,}}_{\textsc {Cov}(N)}(v) =1$, one easily verifies that there is a path P=v⇝u in $\textsc {Cov}(N)$ where *u* satisfies ${{\,\textrm{outdeg}\,}}_{\textsc {Cov}(N)}(u)\ne 1$, and where each vertex *w* along *P* distinct from *u* (if there is one) satisfies $w\in \mathfrak {dvis}(N)$. Clearly, ${{\,\mathrm{\texttt{C}}\,}}_{\textsc {Cov}(N)}(v)={{\,\mathrm{\texttt{C}}\,}}_{\textsc {Cov}(N)}(u)$ and $u\notin \mathfrak {dvis}(N)$. By Lemma 4.2, the vertices in *X* remain vertices in $\textsc {Cov}(N)$. This and Eq. (1) implies that, for each x∈X, we have $x\preceq _{\textsc {Cov}(N)} u$ if and only if $x\preceq _N u$. Consequently, ${{\,\mathrm{\texttt{C}}\,}}_{\textsc {Cov}(N)}(u)={{\,\mathrm{\texttt{C}}\,}}_N(u)$. Similarly, ${{\,\mathrm{\texttt{C}}\,}}_{\textsc {Cov}(N)}(v)={{\,\mathrm{\texttt{C}}\,}}_N(v)$. Since, additionally, $u\prec _N v$, we have shown that *v* has a proper descendant *u* in *N* with the same cluster as *v*. Consequently, $v\notin \operatorname {LCA}_N({{\,\mathrm{\texttt{C}}\,}}_N(v))$ must hold, and this together with (Lindeberg and Hellmuth, 2025, Lem 3.4) implies that $v\notin \operatorname {LCA}_N(A)$ and, in particular, $v\ne \operatorname {lca}_N(A)$ for all non-empty subsets A⊆X. As the latter holds for all vertices $v\in \mathfrak {dvis}(N)$, we conclude that $\mathfrak {dvis}(N)\subseteq \overline{\mathfrak {lca}_{|X|}}(N)$. By Lemma 3.1, we thus have $\mathfrak {dvis}(N)\subseteq \overline{\mathfrak {lca}_{i}}(N)$ for all i≥2. □

By definition, suppression can be formulated in terms of the ⊖-operator (c.f. the discussion right after Definition 2.5), hence the following is an immediate result of Lemma 4.2 and the definition of $\textsc {Norm}(N)$.

### Theorem 4.4

For all networks *N* we have $\textsc {Norm}(N)=\textsc {Cov}(N)\ominus \mathfrak {dvis}(N)=(N\ominus \overline{\mathfrak {vis}}(N))^-\ominus \mathfrak {dvis}(N)$.

**Fig. 9:**
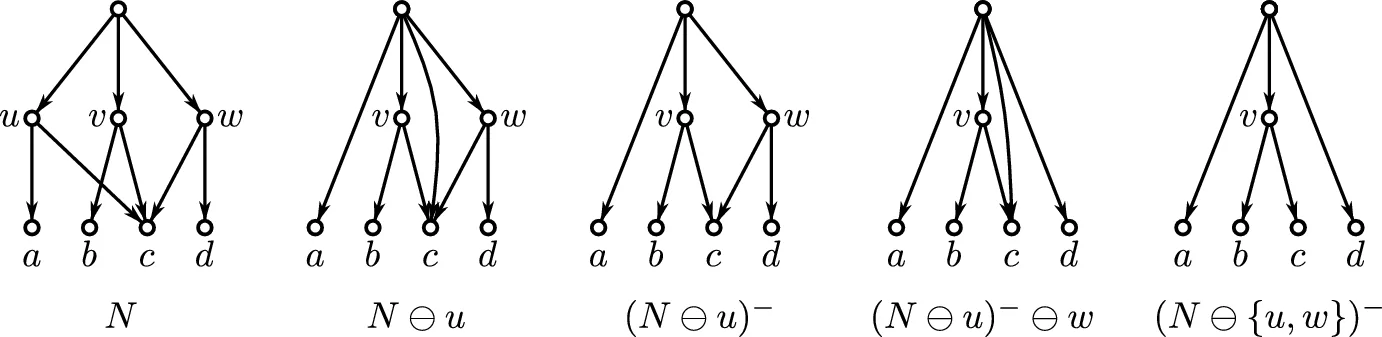
Some networks resulting from the *N* on the very left after application of the ⊖-operator and removal of shortcuts. In particular, note that $(N\ominus u)^-\ominus w$ is not isomorphic to $(N\ominus \{u,w\})^-$

Note that $(N\ominus U)\ominus W = N\ominus (U\cup W)$ always holds by Lemma 2.6. However, the ⊖-operator could result in networks N⊖U that contain shortcuts even if the original network *N* is shortcut-free; for example, see the network N⊖u in Fig. 9. Hence, in general, $(N\ominus U)^-\ominus W\ne (N\ominus (U\cup W))^-$, since $(N\ominus U)^-\ominus W$ may contain shortcuts that, clearly, do not appear in $(N\ominus (U\cup W))^-$; see Fig. 9 again for an example. Intriguingly, as we shall see in Theorem 4.6, $\textsc {Norm}(N)=(N\ominus (\overline{\mathfrak {vis}}(N)\cup \mathfrak {dvis}(N)))^-$. In order to prove this theorem and for completeness, we now show that Theorem 3.3 of Francis et al. (2021) also holds for the more general setting of networks that are not necessarily phylogenetic and separated.

### Proposition 4.5

Let *N* be a network on *X*. The following statements hold.

1. $\textsc {Norm}(N)$ is a normal network on *X* for which $V(\textsc {Norm}(N))=\mathfrak {vis}(N){\setminus } \mathfrak {dvis}(N) = V(N){\setminus } (\overline{\mathfrak {vis}}(N)\cup \mathfrak {dvis}(N))$.
2. For all $$u,v\in V(\textsc {Norm}(N))$$, $$v\preceq _{\textsc {Norm}(N)} u \iff v\preceq _N u$$.
3. If $$v\in V(\textsc {Norm}(N))$$, then $${{\,\mathrm{\texttt{C}}\,}}_{\textsc {Norm}(N)}(v)={{\,\mathrm{\texttt{C}}\,}}_N(v)$$.
4. No vertex *v* of $\textsc {Norm}(N)$ satisfy ${{\,\textrm{indeg}\,}}_{\textsc {Norm}(N)}(v)={{\,\textrm{outdeg}\,}}_{\textsc {Norm}(N)}(v)=1$. In particular, $\textsc {Norm}(N)$ is phylogenetic if and only if its root has out-degree distinct from 1.
5. $\textsc {Norm}(N)=N$ if and only if *N* is normal and there exists no v∈V(N) such that ${{\,\textrm{indeg}\,}}_N(v)={{\,\textrm{outdeg}\,}}_N(v)=1$.
6. If *N* is separated, then $\textsc {Norm}(N)$ is separated.
7. $\textsc {Norm}(N)$ is a tree if and only if $\{{{\,\mathrm{\texttt{C}}\,}}_N(v)\mid v\in \mathfrak {vis}(N)\}$ is a hierarchy.

### Proof

Since the proof is straightforward and resembles (Francis et al., 2021, Thm. 3.3), we have placed it in Appendix A. □

We are now in the position to extend Theorem 4.4 and to characterize those networks *N* for which $\textsc {Norm}(N)$ and $\textsc {Reg}(N)$ coincide.

### Theorem 4.6

Let *N* be a network. Then $\textsc {Norm}(N)=(N\ominus (\overline{\mathfrak {vis}}(N)\cup \mathfrak {dvis}(N)))^-$. Moreover, for each i≥2, we have

2 $$\begin{aligned}&V(\textsc {Reg}_{i}(N)) \cap V(\textsc {Norm}(N)) &  = \mathfrak {lca}_{i}(N) \cap \mathfrak {vis}(N) \end{aligned}$$

3 $$\begin{aligned}&V(\textsc {Reg}_{i}(N)) \setminus V(\textsc {Norm}(N)) &  = \mathfrak {lca}_{i}(N) \setminus \mathfrak {vis}(N) \end{aligned}$$

4 $$\begin{aligned}&V(\textsc {Norm}(N)) \setminus V(\textsc {Reg}_{i}(N)) &  =\mathfrak {vis}(N) \setminus (\mathfrak {lca}_{i}(N)\cup \mathfrak {dvis}(N)). \end{aligned}$$

In particular, $\textsc {Norm}(N)=\textsc {Reg}_{i}(N)$ if and only if $\mathfrak {lca}_{i}(N) = \mathfrak {vis}(N){\setminus } \mathfrak {dvis}(N)$ and, therefore, $\textsc {Norm}(N)=\textsc {Reg}(N)$ if and only if $\mathfrak {lca}_{|X|}(N) = \mathfrak {vis}(N){\setminus } \mathfrak {dvis}(N)$.

### Proof

Let *N* be a network on *X*. For simplicity, put $$N':=(N\ominus (\overline{\mathfrak {vis}}(N)\cup \mathfrak {dvis}(N)))^-$$. By definition of $$\overline{\mathfrak {vis}}(N)$$ and $$\mathfrak {dvis}(N)$$, $$X\subseteq V(N){\setminus } (\overline{\mathfrak {vis}}(N)\cup \mathfrak {dvis}(N))$$. This together with Observation 2.13 ensures that $N^{\prime }$ is a shortcut-free DAG on *X* such that $$V(N')=(V(N){\setminus } \overline{\mathfrak {vis}}(N)){\setminus } \mathfrak {dvis}(N)=\mathfrak {vis}(N){\setminus } \mathfrak {dvis}(N)$$. In addition, Proposition 4.5 shows, in particular, that $$\textsc {Norm}(N)$$ is a shortcut-free network on *X* with $$V(\textsc {Norm}(N))=\mathfrak {vis}(N){\setminus } \mathfrak {dvis}(N)$$. To summarize so far, the graphs $$\textsc {Norm}(N)$$ and $N^{\prime }$ have the same set of vertices and are both shortcut-free. Moreover, by Observation 2.13, we have $$u\preceq _{N'} v$$ if and only if $$u\preceq _N v$$ for all $u,v\in V(N^{\prime })$. By Proposition 4.5, $$u\preceq _{\textsc {Norm}(N)} v$$ if and only if $$u\preceq _N v$$ for all $$u,v\in V(\textsc {Norm}(N))$$. Combining the latter two arguments allow us to conclude that, for all $$u,v\in \mathfrak {vis}(N)\setminus \mathfrak {dvis}(N)$$, we have $$u\preceq _{N'} v$$ if and only if $$u\preceq _{\textsc {Norm}(N)} v$$. By Lemma 2.1, we thus have $$\textsc {Norm}(N)=N'=(N\ominus (\overline{\mathfrak {vis}}(N)\cup \mathfrak {dvis}(N)))^-$$.

Now, fix some integer i≥2. By definition, we have $V(\textsc {Reg}_i(N))= \mathfrak {lca}_{i}(N)$. By Proposition 4.5, $V(\textsc {Norm}(N))=\mathfrak {vis}(N)\setminus \mathfrak {dvis}(N)$. In addition, Lemma 4.3 implies that $\mathfrak {dvis}(N)\subseteq \overline{\mathfrak {lca}_{i}}(N)$ and, therefore, $\mathfrak {lca}_{i}(N)\cap \mathfrak {dvis}(N) = \emptyset$. It now readily follows that $V(\textsc {Norm}(N)) \cap V(\textsc {Reg}_i(N)) = \mathfrak {vis}(N)\cap \mathfrak {lca}_{i}(N)$. Hence, Equation (2) holds. Furthermore, $V(\textsc {Reg}_i(N))\setminus V(\textsc {Norm}(N)) = \mathfrak {lca}_{i}(N) \setminus (\mathfrak {vis}(N)\setminus \mathfrak {dvis}(N))$. Since $\mathfrak {lca}_{i}(N)\cap \mathfrak {dvis}(N) = \emptyset$, the latter simplifies to $V(\textsc {Reg}_i(N)){\setminus } V(\textsc {Norm}(N)) = \mathfrak {lca}_{i}(N) {\setminus } \mathfrak {vis}(N)$, i.e., Equation (3) holds. Moreover, $V(\textsc {Reg}_i(N))= \mathfrak {lca}_{i}(N)$ and $V(\textsc {Norm}(N))=\mathfrak {vis}(N){\setminus } \mathfrak {dvis}(N)$ implies that Equation (4) holds.

To see that the last statement in the theorem holds, first assume that $\textsc {Norm}(N)=\textsc {Reg}_i(N)$. Since $V(\textsc {Norm}(N))=\mathfrak {vis}(N){\setminus } \mathfrak {dvis}(N)$ and $V(\textsc {Reg}_i(N))=\mathfrak {lca}_{i}(N)$, we must in particular have $\mathfrak {vis}(N)\setminus \mathfrak {dvis}(N)=V(\textsc {Norm}(N))=V(\textsc {Reg}_i(N))=\mathfrak {lca}_{i}(N)$. Conversely, assume that $\mathfrak {lca}_{i}(N) = \mathfrak {vis}(N)\setminus \mathfrak {dvis}(N)$. Recall that $\textsc {Norm}(N)=(N\ominus (\overline{\mathfrak {vis}}(N)\cup \mathfrak {dvis}(N)))^-$ and so

$$\begin{aligned} \textsc {Reg}_i(N) = (N\ominus \overline{\mathfrak {lca}_{i}}(N))^-&= (N\ominus (V\setminus \mathfrak {lca}_{i}(N)))^- \\ &= (N \ominus (V\setminus (\mathfrak {vis}(N)\setminus \mathfrak {dvis}(N))))^- \\ &= (N \ominus ((V\setminus \mathfrak {vis}(N)) \cup (V \cap \mathfrak {dvis}(N))))^- \\ &= (N \ominus (\overline{\mathfrak {vis}}(N) \cup \mathfrak {dvis}(N)))^- = \textsc {Norm}(N). \end{aligned}$$

The latter, in particular, shows that $\mathfrak {lca}_{i}(N) = \mathfrak {vis}(N){\setminus } \mathfrak {dvis}(N)$ implies that $\textsc {Reg}_i(N) = \textsc {Norm}(N)$. Hence setting i=|X| implies that $\textsc {Norm}(N)=\textsc {Reg}(N)$ if and only if $\mathfrak {lca}_{|X|}(N) = \mathfrak {vis}(N){\setminus } \mathfrak {dvis}(N)$. □

### Corollary 4.7

For a network *N* and an integer i≥2, the following statements are equivalent.

1. $$\textsc {Norm}(N)=\textsc {Reg}_i(N)$$
2. $$\mathfrak {vis}(N)\setminus \mathfrak {dvis}(N)=\mathfrak {lca}_{i}(N)$$
5. $$\mathfrak {lca}_{i}(N)\cap \overline{\mathfrak {vis}}(N)=\emptyset $$ and $$\overline{\mathfrak {lca}_{i}}(N)\cap \mathfrak {vis}(N)=\mathfrak {dvis}(N)$$.

### Proof

Since the proof is tedious but straightforward, we have placed it in Appendix A. □

### Corollary 4.8

Given a network *N*, testing if $\textsc {Reg}(N)=\textsc {Norm}(N)$ can be done in polynomial time in |*V*(*N*)|.

### Proof

Let *N* be a network on *X*. As outlined in the appendix of Francis et al. (2021), $\textsc {Norm}(N)$ can be computed in polynomial time, whenever the network *N* is separated. In fact, the exact same approach may be used to compute $\textsc {Norm}(N)$ for a network that is not necessarily separated which, in particular, provides access to the set $V(N)=\mathfrak {vis}(N)\setminus \mathfrak {dvis}(N)$. As outlined in the proof of Proposition 3.9, the set $\overline{\mathfrak {lca}_{|X|}}(N)$ and, thus, $\mathfrak {lca}_{|X|}(N)= V(N)\setminus \overline{\mathfrak {lca}_{|X|}}(N)$ can be computed in polynomial time in |*V*(*N*)|. Since, by Theorem 4.6, $\textsc {Norm}(N)=\textsc {Reg}(N)$ if and only if $\mathfrak {lca}_{|X|}(N) = \mathfrak {vis}(N)\setminus \mathfrak {dvis}(N)$, we can thus test in polynomial time whether $\textsc {Norm}(N)=\textsc {Reg}(N)$ by a straightforward set comparison in $O(|V(N)|^2)$ time. □

## Discussion and Outlook

In this paper, we have introduced and explored a framework for simplifying DAGs, where the simplified DAGs have the form $(G\ominus W)^-$ for certain subsets *W* of the vertices of *G*. To be more precise, in Sect. 3, we studied the *i*-regularization $\textsc {Reg}_i(G)$ of a DAG *G*, where *W* is the set $\overline{\mathfrak {lca}_{i}}(G)$ of vertices that are not the unique least common ancestor of any subset of leaves of size at most *i*. In particular, we noted that $\textsc {Reg}_i(G)$ is both *i*-lca-relevant and regular (c.f. Theorem 3.3). We then proceeded to describe the clusters of $\textsc {Reg}_i(G)$ in Theorem 3.5 which, due to regularity, fully determine the structure of $\textsc {Reg}_i(G)$. In Sect. 3.2, we studied regularizations of normal networks, in particular showing that regularizations preserve the property of being a normal network (Theorem 3.14). Finally, in Sect. 4, we studied the normalization $\textsc {Norm}(N)$ of a network *N* and showed, in Theorem 4.6, that $\textsc {Norm}(N)=(N\ominus W)^-$ for $W=\overline{\mathfrak {vis}}(N)\cup \mathfrak {dvis}(N)$. In other words, the set *W* contains all vertices of *N* that are not visible and all vertices of *N* that are visible but have in- and out-degree 1 in $\textsc {Cov}(N)$ (where the latter vertices, in particular, can be determined by properties in *N* itself, see Lemma 4.3). Moreover, Theorem 4.6 also characterizes those networks *N* for which $\textsc {Norm}(N)$ and $\textsc {Reg}_i(N)$ coincide.

The comparison between regularization and normalization also highlights that neither simplification is uniformly more informative than the other. Their behavior depends on which type of leaf-based information is regarded as relevant. There are regular networks for which regularization retains essentially the entire structure while normalization retains only very little, since most vertices are not visible. Conversely, there are networks for which normalization retains substantial structure although few, or no, internal vertices occur as unique LCAs of subsets of leaves, so that regularization removes most of the network (refer back to Fig. 8, from which larger examples can easily be obtained). Thus, the two approaches should be viewed as complementary rather than competing simplification procedures. This difference also comes with a potential complexity trade-off. Regular DAGs may in general contain many more vertices than normal or strongly normal networks (see, for example, (Willson, 2010, Thm. 5.1)), and hence a regularization that retains a large collection of LCA-induced clusters may be substantially more complex than a corresponding normalization. On the other hand, such additional vertices may be relevant when they are supported by the available data, for example through clusters or inferred LCA relationships. Which simplification is preferable therefore depends not only on structural considerations but also on the type of information that can be reliably inferred.

It could be worthwhile to investigate how different choices of subsets *W* affect the simplification $(G\ominus W)^-$ of a given DAG or network *G*. By the results developed in Lindeberg and Hellmuth (2025), cf. Observation 2.13, the DAG $H:=(G\ominus W)^-$ always preserves the $\preceq _G$-relation i.e. $u\preceq _{H}v$ if and only if $u\preceq _G v$ for all u,v∈V(H). Consequently, $\mathfrak {C}(H)\subseteq \mathfrak {C}(G)$, provided that *W* contains no leaves of *G*. Thus, the simplification *H* never introduces clusters that were not already present in *G*.

In another more general direction, in (Francis, 2025, p.4), Francis states

“The normalization captures the key information in *any* given network: the information that is in fact reconstructable, that is, the vertices and edges whose evolutionary signal may be visible in the leaves. If one has data that evolved from a process on a network, reconstructing the normalization of the network is the sweet spot: both the most information reasonable to hope for, and the most that can practically be obtained.”

This provides a compelling argument for the normalization procedure in particular, and for normal networks more generally. Although normalization is designed to remove vertices that are not visible, visibility is a path-based notion of relevance that depends on the topology of the entire network. From the perspective of data-driven simplification, it may therefore not be always clear which observable data would directly support such a topological property, especially when the underlying network is unknown. For example, depending on the data at hand, it is conceivable that one may be able to infer the existence of a vertex *v* with a particular cluster $C={{\,\mathrm{\texttt{C}}\,}}_N(v)$, even if no vertex *u* with ${{\,\mathrm{\texttt{C}}\,}}_N(u)=C$ is visible in the network *N*. Such a cluster would disappear under normalization, since no vertex with cluster *C* would be retained; cf. Proposition 4.5(3).

In general, such considerations could be very important when trying to take into account the data when simplifying networks. For example, there is evidence that relationships between LCAs can, to some extent, be inferred from so-called best hits (Geiß et al. 2020; Stadler et al. 2020). More precisely, pairwise similarities between genomic sequences associated with leaves *a*, *b*, and $b^{\prime }$, where *a* belongs to some species *A* and $b,b^{\prime }$ belong to a species *B* distinct from *A*, may support conclusions of the form $$\operatorname {lca}_N(a,b)\preceq _N\operatorname {lca}_N(a,b')$$ even without prior knowledge of the structure of the underlying network *N*. The vertices $$\operatorname {lca}_N(a,b)$$ and $$\operatorname {lca}_N(a,b')$$ need not be visible, however, and may therefore be removed by the normalization $$\textsc {Norm}(N)$$. Thus, it may be useful to consider more general simplifications of the form $$(N\ominus W)^-$$, where *W* need not contain all non-visible vertices. Such a choice of *W* allows one to retain non-visible vertices whose relevance is nevertheless supported by the available data, for instance because they induce observed clusters or occur as unique LCAs of pairs, or more generally subsets, of leaves.

In summary, the main point is that, depending on the type of data under consideration, there may be different notions of what constitutes the *key information* of a network and, consequently, of what makes a vertex relevant for simplification. Thus, network simplifications should aim to retain those vertices whose existence is supported, or witnessed, by the available data. If the relevant signal is visibility, then this requires information about paths from vertices to leaves. If the data are clusters, then vertices *v* with ${{\,\mathrm{\texttt{C}}\,}}_N(v)$ among the supported clusters should be retained, since their relevance is witnessed by the cluster system. Similarly, if the data support the existence or relative position of LCAs of pairs of leaves, then the corresponding LCA vertices should be retained. In this sense, the appropriate simplification of a network should be guided by the kind of evolutionary signal present in the data, rather than by a single universal notion of vertex relevance and, indeed, this information could also be useful for the more challenging problem of constructing a network in the first place.

## Data Availability

No data sets are associated with this manuscript.

## A Outsourced Proofs

### Proof of Lemma 2.6

Let *G* be a DAG and u,v∈V(G) be distinct vertices. By definition, the vertex sets of $(G \ominus v) \ominus u$ and $(G \ominus u) \ominus v$ coincide. We show now that their arc sets coincide. To this end, let p→q be an arc of $(G \ominus v) \ominus u$ and thus, $\{p,q\}\cap \{u,v\}=\emptyset$ and, in particular, $|\{p,q, u,v\}|=4$. We will show that p→q is an arc of $(G\ominus u)\ominus v$.

If (p,q)∈E(G) then it is, by definition, also an arc in $(G \ominus u) \ominus v$. Suppose now that (p,q)∉E(G). Now, one of the following cases occurs.

**Case: **$(p,q)\in E(G\ominus v)$.: Since (p,q)∉E(G), it holds that $p\in {{\,\textrm{par}\,}}_{G}(v)$ and $q\in {{\,\textrm{child}\,}}_{G}(v)$. Since p,q,v≠u, the arcs p→v and v→q remain in G⊖u i.e. $p\in {{\,\textrm{par}\,}}_{G\ominus u}(v)$ and $q\in {{\,\textrm{child}\,}}_{G\ominus u}(v)$. Thus, by definition, $(p,q)\in E((G \ominus u) \ominus v)$.
**Case: **$(p,q)\notin E(G\ominus v)$.: In this case, $(p,q)\in E((G \ominus v) \ominus u)\setminus E(G\ominus v)$ hold. Hence, by definition, $p\in {{\,\textrm{par}\,}}_{G\ominus v}(u)$ and $q\in {{\,\textrm{child}\,}}_{G\ominus v}(u)$. In other words, $(p,u),(u,q)\in E(G\ominus v)$. Now one of the following subcases occurs. **Subcase:**(p,u),(u,q)∈E(G).: By definition, $(p,q) \in E(G\ominus u)$. Since, p,q≠v it follows that $(p,q) \in E((G\ominus u)\ominus v)$. **Subcase: **$(p,u)\notin E(G),(u,q)\in E(G)$.: Since (*p*, *u*) is an arc of G⊖v but not of *G*, the arcs p→v and v→u must exist in *G*. Since also u→q is an arc of *G*, it follows that there is a path $p\rightarrow v\rightarrow u\rightarrow q$ in *G*. By definition, p→v and v→q are arcs of G⊖u, and it follows that p→q is an arc of $(G\ominus u)\ominus v$. **Subcase:**$(p,u)\in E(G),(u,q)\notin E(G)$.: Since u→q is an arc of G⊖v but not of *G*, u→v and v→q must be arcs of *G*. Thus, there is a path $p\rightarrow u\rightarrow v\rightarrow q$ in *G*. By similar arguments as in the previous case, p→q is an arc of $(G\ominus u)\ominus v$. **Subcase:**(p,u),(u,q)∉E(G).: As argued before, since $(p, u)\in E(G\ominus v)$ and (p,u)∉E(G), the arc v→u must exist in *G*. Moreover, since $(u, q) \in E(G\ominus v)$ and (u,q)∉E(G), u→v is an arc of *G*. The existence of the two arcs u→v and v→u in *G*, however, contradicts the fact that *G* is a DAG. Hence, this case cannot occur.

In summary, in all cases that can occur, $(p,q)\in E((G\ominus v)\ominus u)$ implies $(p,q)\in E((G\ominus u)\ominus v)$. By analogous argumentation, $E((G\ominus u)\ominus v)\subseteq E((G\ominus v)\ominus u)$ holds and thus, the arc sets of the graphs coincide. Therefore, $(G \ominus v) \ominus u = (G \ominus u) \ominus v$. □

### Proof of Lemma 3.10

Let *G* be a strongly normal DAG on *X*. If *G* has k≥2 roots $r_1,\ldots ,r_k$, we construct the network *N* by adding a new root ρ and the arc $\rho \rightarrow r_i$ for each $1\le i\le k$. If *G*, instead, is already a network, we put N:=G and let ρ denote its unique root. It is easy to see that in both cases, N=(V,E) is a strongly normal network on *X*. We will now use (Willson, 2010, Thm 3.9) established by Willson to prove the result. To this end, we define the *base-set* ${\widetilde{X}}_N$ of *N* as the set of vertices in *N* comprising the root, the leaves, and all vertices with out-degree 1. In our setting ${\widetilde{X}}_N = X\cup \{\rho \}$, since strongly normal networks by definition have no vertices with out-degree 1. Willson has shown that each vertex $v\in V\setminus {\widetilde{X}}_N$ satisfies $v=\operatorname {lca}_N(x,y)$ for some $x,y\in {\widetilde{X}}_N$. Note that $\operatorname {lca}_N(\{x\})=x$ for all x∈X. By the latter arguments, all vertices $v\in V \setminus \{\rho \}$ satisfy $v=\operatorname {lca}_N(x,y)$ for some $x,y\in X\cup \{\rho \}$. As $v\prec _N \rho$ for all $v\in V {\setminus } \{\rho \}$, we obtain $v=\operatorname {lca}_N(x,y)$ for some x,y∈X.

Hence, if *G* has multiple roots, then the latter result together with the fact that $u\preceq _G v$ if and only if $u\preceq _N v$ for all $u,v\in V(G)=V{\setminus } \{\rho \}$ implies that, for all v∈V(G), there are some x,y∈X such that $v=\operatorname {lca}_N(x,y)$. Therefore, *G* is 2-lca-relevant in case G≠N.

We now consider the case when N=G. Hence, in order to show that *N* is 2-lca-relevant, it remains to show that $\rho =\operatorname {lca}_N(x,y)$ for some x,y∈X. To this end, we extend *N* by adding a vertex *r* and the arc $r\rightarrow \rho$, resulting in the network $N^*$. It is easy to verify that $N^*$ remains a normal network on *X* with base-set ${\widetilde{X}}_{N^*} = X\cup \{r\}$. We can now apply (Willson, 2010, Thm 3.9) again to conclude that $\rho =\operatorname {lca}_{N^*}(x,y)$ for some $x,y\in X\cup \{r\}$. By definition of least common ancestors we have $x,y\preceq _{N^*}\operatorname {lca}_{N^*}(x,y)=\rho \prec _{N^*} r$, which ensures that r≠x and r≠y. In other words, $\rho =\operatorname {lca}_{N^*}(x,y)$ implies x,y∈X. It is now straight-forward to check that $\rho =\operatorname {lca}_{N}(x,y)$ holds. In summary, *G* is 2-lca-relevant, in the case that G=N.

Thus, *G* is 2-lca-relevant in both cases. In particular, *G* is *k*-lca-relevant for all $k\in \{2,\dots ,|X|\}$. □

### Proof of Proposition 3.12

Let G=(V,E) be a normal DAG on *X* and put $U :=\{v\in V\mid {{\,\textrm{outdeg}\,}}_G(v)=1\}$. Let v∈U and denote with *c* the unique child of *v*. By Lemma 2.7, G⊖v is a DAG on *X*. We start with showing that G⊖v remains normal. Since *G* is tree-child and since *c* is the unique child of *v* it must be a tree-vertex, i.e., ${{\,\textrm{indeg}\,}}_G (c) = 1$, which implies that *v* is the unique parent of *c*.

We continue with showing that G⊖v remains tree-child. Let $u\in V(G\ominus v){\setminus } X$. We consider now the following cases for *u*: (i) u=c and (ii) *u* is a parent of *v* and (iii) u≠c and *u* is not a parent of *v*.

Case (i): u=c. Since *G* is tree-child and u∉X, u=c has a tree-child $c^{\prime }$ in *G*. Since the children of *c* in G⊖v are precisely the children of *c* in *G* it follows that $c^{\prime }$ is a child of *c* in G⊖v. Moreover, as *v* is the unique parent of *c* and u≠v, it follows that $c^{\prime }$ is not a child of (and since *G* is a DAG also no parent of) *v*. Hence, the out-degree and in-degree of $c^{\prime }$ in *G* is the same as in G⊖v, and we can conclude that $c^{\prime }$ remains a tree-child of u=c in G⊖v.

Case (ii): *u* is a parent of *v* in *G*. If *u* is the unique parent of *v* in *G*, then *u* is the unique parent of *c* in G⊖v, in which case *c* becomes the new tree-child of *u* in G⊖v. If *u* is one of at least two parents, then *v* is not a tree-child of *u* in *G* since $${{\,\textrm{indeg}\,}}_G (v) > 1$$. However, *u* must have a tree-child $c^{\prime }$ in *G*. As *v* is the unique parent of *c* it follows that $c\neq c^{\prime }$ and, therefore, $c^{\prime }$ cannot be a child of *v* in *G*. Moreover, $c^{\prime }$ cannot be a parent of *v* in *G* as otherwise the arc u→v would be a shortcut. Hence, the out-degree and in-degree of $c^{\prime }$ in *G* is the same as in G⊖v, and we can conclude that $c^{\prime }$ remains a tree-child of *u* in G⊖v.

Case (iii): *u* is neither a parent nor a child of *v*. Since *G* is tree-child, *u* must have a tree-child $c^{\prime }$ in *G*. Since *u* is not a parent of *v*, it follows that $c^{\prime }\neq v$ and, therefore, $$c'\in V(G\ominus v)$$. Since u≠v and *v* is the unique parent of *c*, we can conclude that $c\neq c^{\prime }$. It immediately follows, by construction of G⊖v that the in-degree of $c^{\prime }$ in *G* and G⊖v remain the same, i.e., $c^{\prime }$ remains a tree-child of *u*. In summary, all vertices $$u\in V(G\ominus v)\setminus X$$ have a tree-child and thus, G⊖v is tree-child.

We continue by showing that G⊖v remains shortcut-free. Assume, for contradiction, that G⊖v contains a shortcut (*a*, *b*). Hence, there is an alternative path P=a⇝b in G⊖v that does not contain the arc (*a*, *b*). Note that $b\prec _{G\ominus v} a$, v∉X and Observation 2.13 implies that $b\prec _G a$ holds. Suppose first that (a,b)∉E(G). The latter is, by construction of G⊖v, only possible if c=a or c=b holds for the unique child *c* of *v*. However, since the children of *c* and their descendants in *G* and G⊖v are identical, a=c is not possible as then (a,b)∈E(G). Thus, c=b must hold, in which case *a* must be a parent of *v* in *G*, i.e., there is an arc a→v in *G*. Since *c* is the unique child of *v* in *G* and *v* the unique parent of *c* in *G*, it follows that any path from *a* to b=c must contain the arc v→b. However, there is the alternative *P* from *a* to *b* in G⊖v. One easily verifies that this is only possible if there is a path $P' = a\leadsto v\rightarrow b$ in *G* where the subpath a⇝v contains more than the single arc a→v. But then *G* is not shortcut-free; a contradiction. Therefore, (a,b)∈E(G) must hold. However, since P=a⇝b is a directed path from *a* to *b* in G⊖v it follows that *v* is not contained in *P* and Observation 2.13 implies that all vertices *w* in *P* satisfy $b\preceq _G w\preceq _G a$. As at least one such vertex w≠a,b exists it follows that there is an alternative path a⇝b in *G*, a contradiction to *G* being shortcut-free. Thus, G⊖v is shortcut-free.

In summary, we have shown that G⊖v is a shortcut-free and tree-child DAG on *X*, i.e., G⊖v is a normal DAG on *X*. Recall that, since *G* is tree-child, the unique child *c* of *v* satisfies ${{\,\textrm{indeg}\,}}_G(c)=1$. Therefore, Lemma 2.14 implies that the out-degrees of vertices in G⊖v are the same in *G* and G⊖v, the previous arguments and induction can be used to show that for all W⊆U it holds that G⊖W is a normal DAG on *X*. As *U* is the set of all vertices of out-degree 1 in *G* and each vertex in *U* has a tree-child, Lemma 2.14 also implies that G⊖U cannot have vertices of out-degree 1. Hence, G⊖U is, in particular, strongly normal.

Finally, we will show that $\overline{\mathfrak {lca}_{i}}(G)=U$ for all i≥2. We first show that $\overline{\mathfrak {lca}_{2}}(G)=U$ by verifying that $V\setminus \overline{\mathfrak {lca}_{2}}(G) = V\setminus U$. Since, by Lemma 3.1, $U\subseteq \overline{\mathfrak {lca}_{2}}(G)$ it follows that $V{\setminus } \overline{\mathfrak {lca}_{2}}(G) \subseteq V{\setminus } U$. Now let $v\in V{\setminus } U$. By definition, $v\in V(G\ominus U)$; see also Lemma 2.7. As shown in the previous paragraph, G⊖U is strongly normal and so Lemma 3.10 implies that G⊖U is 2-lca-relevant. Hence, there are leaves x,y∈X such that $v = \operatorname {lca}_{G\ominus U}(x,y)$. In other words, $\operatorname {LCA}_{G\ominus U}(\{x,y\})=\{v\}$. This together with Lemma 2.14 implies that $\operatorname {LCA}_{G}(\{x,y\})=\{v\}$ and, therefore, $v = \operatorname {lca}_G(x,y)$. By definition, $v\in \mathfrak {lca}_{2}(G)=V{\setminus } \overline{\mathfrak {lca}_{2}}(G)$. In conclusion, $U = \overline{\mathfrak {lca}_{2}}(G)$ holds. This together with Lemma 3.1 implies that $\overline{\mathfrak {lca}_{i}}(G)=U$ for all integers i≥2. □

### Proof of Lemma 4.2

Let *N* be a network on *X* with unique root ρ. Let *G* denote the digraph as specified in Definition 4.1(1). Hence, $\textsc {Cov}(N)=G^-$. Since $V(G)=\mathfrak {vis}(N)$ and u→v is an arc of *G* if and only if $v\prec _N u$, it follows that there is a directed *uv*-path in *G* if and only if $u,v\in \mathfrak {vis}(N)$ and $v\preceq _N u$. Consequently, *G* is a DAG such that

5 $$\begin{aligned} v\preceq _{G} u \iff v\preceq _N u,\quad \text { for all }u,v\in \mathfrak {vis}(N). \end{aligned}$$

Note that, by definition of visibility, $\rho \in \mathfrak {vis}(N)$ and $X\subseteq \mathfrak {vis}(N)$. Moreover, for all *v* in *N*, $v\preceq _N \rho$ and there is a leaf x∈X with $x\preceq _N v$. The latter two arguments together with Eq. (5) ensure that ρ remains the unique root of *G* and that the set of leaves of *G* is *X* and, in particular, that *G* is network on *X*

Now, by definition, $\textsc {Cov}(N)=G^-$ and $\textsc {Cov}(N)$ is, by Lemma 2.1 and the arguments in the previous paragraph, a shortcut-free network on *X* which satisfy $V(\textsc {Cov}(N))=V(G)=\mathfrak {vis}(N)$. Moreover, Lemma 2.1 together with Eq. (5) also ensures that

6 $$\begin{aligned} v\preceq _{\textsc {Cov}(N)} u \iff v\preceq _N u,\quad \text { for all }u,v\in \mathfrak {vis}(N). \end{aligned}$$

Now consider $N':=(N\ominus \overline{\mathfrak {vis}}(N))^-$. Since $\overline{\mathfrak {vis}}(N)\subseteq V(N){\setminus } X$, we can apply Lemma 2.1 and Lemma 2.7 to conclude that $V(N')=V(N)\setminus \overline{\mathfrak {vis}}(N)=\mathfrak {vis}(N)$ and that

7 $$\begin{aligned} v\preceq _N u \iff v\preceq _{N'} u,\quad \text { for all }u,v\in \mathfrak {vis}(N). \end{aligned}$$

By, Lemma 2.1, $N^{\prime }$ is shortcut-free. We have thus shown that $N^{\prime }$ and $$\textsc {Cov}(N)$$ are shortcut-free DAGs with common vertex set $$\mathfrak {vis}(N)$$. Moreover, Eqs. (6) and (7) together shows that $$u\preceq _{\textsc {Cov}(N)} v$$ if and only if $$u\preceq _{N'} v$$ for all $$u,v\in \mathfrak {vis}(N)$$. Thus Lemma 2.1 implies that $$\textsc {Cov}(N)=N'=(N\ominus \overline{\mathfrak {vis}}(N))^-$$.

Finally, we show that $\textsc {Cov}(N)$ is normal network on *X*. Recall that $\textsc {Cov}(N)$ is a network on *X*. We continue by showing that every vertex of $\textsc {Cov}(N)$ is visible. Assume, for contradiction, that there is a vertex *v* in $\textsc {Cov}(N)$ that is not visible. Let x∈X be a leaf with $x\preceq _N v$ and $P_1 = \rho \leadsto v\leadsto x$ be directed path in $\textsc {Cov}(N)$. Since, *v* is not visible in $\textsc {Cov}(N)$ there is an alternative path $P_2 = \rho \leadsto x$ that does not contain *v*. By Eq. (6), there are paths $P'_1 = \rho \leadsto v\leadsto x$ and $P'_2 = \rho \leadsto x$ in *N* where $P'_2$ does not contain *v*. Hence, *v* is not visible in *N*; a contradiction to $v\in V(\textsc {Cov}(N))=\mathfrak {vis}(N)$. Thus, all vertices of $\textsc {Cov}(N)$ are visible. Since $\textsc {Cov}(N) = G^-$ is a shortcut-free network, Theorem 2.4 implies that $\textsc {Cov}(N)$ is normal. □

### Proof of Proposition 4.5

Let *N* be a network on *X* and consider $\textsc {Norm}(N)$. By Theorem 4.4, $\textsc {Norm}(N)=\textsc {Cov}(N)\ominus \mathfrak {dvis}(N)=(N\ominus \overline{\mathfrak {vis}}(N))^-\ominus \mathfrak {dvis}(N)$. By definition, $\textsc {Cov}(N)$ has vertex set $\mathfrak {vis}(N)$ and, by Lemma 4.2, $\textsc {Cov}(N)$ is a normal network on *X*. Furthermore, by definition, $\mathfrak {dvis}(N)\subseteq \{v\in V(\textsc {Cov}(N))\mid {{\,\textrm{outdeg}\,}}_{\textsc {Cov}(N)}(v)=1\}$. The latter two facts together with Proposition 3.12 ensure that $\textsc {Cov}(N)\ominus \mathfrak {dvis}(N)$ is a normal DAG on *X*. Since, by Theorem 4.4, $\textsc {Norm}(N)=\textsc {Cov}(N)\ominus \mathfrak {dvis}(N)$, it follows that $\textsc {Norm}(N)$ is a normal DAG on *X*. In particular, Lemma 2.7 ensures that $V(\textsc {Norm}(N))=V(\textsc {Cov}(N))\setminus \mathfrak {dvis}(N)=\mathfrak {vis}(N)\setminus \mathfrak {dvis}(N)$. In addition, Theorem 4.4 implies that $V(\textsc {Norm}(N))=V(N)\setminus (\overline{\mathfrak {vis}}(N)\cup \mathfrak {dvis}(N))$, and Lemma 2.7 and Eq. (6) together ensure that

$$\begin{aligned} v\preceq _{\textsc {Norm}(N)} u \iff v\preceq _{\textsc {Cov}(N)} u \iff v\preceq _{N} u\quad \text { for all }u,v\in V(\textsc {Norm}(N))\subseteq V(\textsc {Cov}(N)). \end{aligned}$$

In particular, the unique root ρ of $\textsc {Cov}(N)$ will, by definition, remain a vertex in $\textsc {Norm}(N)$ and satisfies $v\preceq _{\textsc {Norm}(N)}\rho$ for all $v\in V(\textsc {Norm}(N))$. Therefore, $\textsc {Norm}(N)$ has a unique root and is indeed a normal *network* on *X*. We have thus proven statement (1) and (2) in the proposition. Statement (3) is an immediate consequence of (2), since for all x∈X and $v\in V(\textsc {Norm}(N))$ we have $x\preceq _N v$ if and only if $x\preceq _{\textsc {Norm}(N)} v$.

For the first part of Statement (4), recall that, by definition, $\textsc {Norm}(N)=\textsc {Cov}(N)\ominus \mathfrak {dvis}(N)$. It is easily verified that if $v\in \mathfrak {dvis}(N)$ i.e. if ${{\,\textrm{indeg}\,}}_{\textsc {Cov}(N)}(v)={{\,\textrm{outdeg}\,}}_{\textsc {Cov}(N)}(v)=1$, then ${{\,\textrm{outdeg}\,}}_{\textsc {Cov}(N)\ominus v}(u)={{\,\textrm{outdeg}\,}}_{\textsc {Cov}(N)}(u)$ and ${{\,\textrm{indeg}\,}}_{\textsc {Cov}(N)\ominus v}(u)={{\,\textrm{indeg}\,}}_{\textsc {Cov}(N)}(u)$ for all $u\in V(\textsc {Cov}(N)\ominus v)$. Consequently, $\textsc {Norm}(N)=\textsc {Cov}(N)\ominus \mathfrak {dvis}(N)$ contains no vertex of in- and out-degree 1. The second part of Statement (4) is an immediate consequence of the first part and the definition of phylogenetic networks.

Next consider Statement (5). The *only-if* part is an immediate consequence of Statement (1) and (4). For the *if* part, suppose that *N* is normal and that there exists no v∈V(N) such that ${{\,\textrm{indeg}\,}}_N(v)={{\,\textrm{outdeg}\,}}_N(v)=1$. By Theorem 2.4, $V(N)=\mathfrak {vis}(N)$, so Lemma 4.2 implies that $\textsc {Cov}(N)=(N\ominus \overline{\mathfrak {vis}}(N))^-=(N\ominus \emptyset )^-=N$, in particular applying that *N* is shortcut-free. Since $\textsc {Cov}(N)=N$ and there is no v∈V(N) such that ${{\,\textrm{indeg}\,}}_N(v)={{\,\textrm{outdeg}\,}}_N(v)=1$, we also have $\mathfrak {dvis}(N)=\emptyset$. Hence, $\textsc {Norm}(N)=\textsc {Cov}(N)\ominus \mathfrak {dvis}(N)=\textsc {Cov}(N)=N$ follows.

The final two statements can be proved verbatim as in the proof of (Francis et al., 2021, Thm. 3.3) (see Appendix A therein) and are therefore left to the reader. □

### Proof of Corollary 4.7

Let N=(V,E) be a network. The equivalence between Condition (1) and (2) has been shown in Theorem 4.6. Suppose Condition (2) holds. Then, $\mathfrak {vis}(N) = (\mathfrak {vis}(N)\setminus \mathfrak {dvis}(N))\cup \mathfrak {dvis}(N)=\mathfrak {lca}_{i}(N)\cup \mathfrak {dvis}(N)$. Since, by Lemma 4.3, $\mathfrak {dvis}(N)\subseteq \overline{\mathfrak {lca}_{i}}(N)$ it follows that $\mathfrak {lca}_{i}(N)$ and $\mathfrak {dvis}(N)$ are disjoint. Taken the latter two arguments together,  and, thus, Condition (3) holds. Suppose Condition (3) holds. Hence, . Note that $V\setminus \mathfrak {vis}(N) = \overline{\mathfrak {vis}}(N)$ and that . Hence, $\overline{\mathfrak {vis}}(N) = \overline{\mathfrak {lca}_{i}}(N) \setminus \mathfrak {dvis}(N)$ and consequently, , i.e., Condition (4) holds. If Condition (4) holds, then . Thus, . By definition, $\textsc {Reg}_i(N)=(N\ominus \overline{\mathfrak {lca}_{i}}(N))^-$ and, by Theorem 4.6, . Therefore, $\textsc {Norm}(N)=\textsc {Reg}_i(N)$ and Condition (1) holds. In summary, Condition (1)–(4) are equivalent.

For the final equivalence, first assume that Condition (3), or equivalently, (4) holds. By Condition (3), . Hence, . Therefore, $\mathfrak {lca}_{i}(N)\cap \overline{\mathfrak {vis}}(N)=\emptyset$. Moreover, by Condition (4),  where the latter equation follows from the fact that, by definition, $\overline{\mathfrak {vis}}(N)\cap \mathfrak {vis}(N)=\emptyset$ and $\mathfrak {dvis}(N)\subseteq \mathfrak {vis}(N)$. Thus, Condition (5) holds. Assume that Condition (5) holds. By definition,  and . Hence, . Since, by assumption, $\mathfrak {lca}_{i}(N)\cap \overline{\mathfrak {vis}}(N)=\emptyset$, we obtain . Moreover, since , we obtain $\overline{\mathfrak {lca}_{i}}(N)\setminus \overline{\mathfrak {vis}}(N)=\overline{\mathfrak {lca}_{i}}(N)\cap \mathfrak {vis}(N)=\mathfrak {dvis}(N)$. Hence, , i.e., Condition (3) holds. □
